# Can anti-parasitic drugs help control COVID-19?

**DOI:** 10.2217/fvl-2021-0160

**Published:** 2022-03-18

**Authors:** Yasin Panahi, Masoomeh Dadkhah, Sahand Talei, Zahra Gharari, Vahid Asghariazar, Arash Abdolmaleki, Somayeh Matin, Soheila Molaei

**Affiliations:** ^1^Department of Pharmacology & Toxicology, School of Pharmacy, Ardabil University of Medical Sciences, Ardabil, Iran; ^2^Pharmaceutical Sciences Research Center, Ardabil University of Medical Sciences, Ardabil, Iran; ^3^School of Medicine, Tehran University of Medical Sciences, Tehran, Iran; ^4^Department of Biotechnology, Faculty of Biological Sciences, Al-Zahra University, Tehran, Iran; ^5^Deputy of Research & Technology, Ardabil University of Medical Sciences, Ardabil, Iran; ^6^Department of Engineering Sciences, Faculty of Advanced Technologies, University of Mohaghegh Ardabili, Namin, Iran; ^7^Bio Science & Biotechnology Research center (BBRC), Sabalan University of Advanced Technologies (SUAT), Namin, Iran; ^8^Department of Internal Medicine, Imam Khomeini Hospital, Ardabil University of Medical Sciences, Ardabil, Iran; ^9^Pharmaceutical Sciences Research Center, Ardabil University of Medical Sciences, Ardabil, Iran; ^10^Zoonoses Research Center, Ardabil University of Medical Sciences, Ardabil, Iran

**Keywords:** anti-parasitic drugs, artificial intelligence, COVID-19, drug repositioning, epidemic, pandemic, SARS-CoV-2

## Abstract

Novel COVID-19 is a public health emergency that poses a serious threat to people worldwide. Given the virus spreading so quickly, novel antiviral medications are desperately needed. Repurposing existing drugs is the first strategy. Anti-parasitic drugs were among the first to be considered as a potential treatment option for this disease. Even though many papers have discussed the efficacy of various anti-parasitic drugs in treating COVID-19 separately, so far, no single study comprehensively discussed these drugs. This study reviews some anti-parasitic recommended drugs to treat COVID-19, in terms of function and *in vitro* as well as clinical results. Finally, we briefly review the advanced techniques, such as artificial intelligence, that have been used to find effective drugs for the treatment of COVID-19.

Over the past 20 years, the world encountered two coronavirus-related epidemics: severe acute respiratory syndrome (SARS), which emerged in China at the end of 2003, and Middle East respiratory syndrome (MERS), which was first reported in Saudi Arabia in 2012. At the end of December 2019, the novel SARS-CoV-2 first appeared in Wuhan, Hubei Province, China, and rapidly spread to around the globe in a few weeks. On 30 January 2020, COVID-19, the disease caused by this virus, was announced a pandemic by the WHO [1]. Similar to previous pathogenic coronaviruses, SARS-CoV-2 causes pneumonia and severe respiratory syndrome [2,3].

The COVID-19 outbreak, the second global pandemic after MERS, has had a devastating impact on not just the healthcare system but also the global economy [4,5]. SARS-CoV-2 is an enveloped and positive-stranded RNA virus transmitted from humans to humans by exposure to respiratory droplets containing viral particles or touching contaminated surfaces. Because of the high inter-transmission rate among humans, physical distancing, wearing a face mask and following health instructions are the best-recommended ways to stop the spread of this disease [6].

During the second year of this pandemic, new variants of the SARS-CoV-2, including Alpha (B.1.1.7), Beta (B.1.351), Gamma (P.1), Kappa, Delta (B.1.617.1 and B.1.617.2) and Delta Plus (AY. 1) were identified and reported across the world. These new variants are named ‘variants of concern’ (VOCs), which have caused global health havoc [7,8]. The Delta variant has attracted significant attention due to a faster transmission rate, especially among children; being more contagious than other variants; causing a more severe illness in patients; and being less responsive to treatment [9]. Despite the resistance of emerging Delta variants to monoclonal antibody cocktail treatments, antibody therapy could still be a possible therapeutic way to control Delta and other VOCs [10]. In this regard, a study conducted in Vero and BEAS-2B cell lines indicated that meplazeumab, a humanized anti-CD147 antibody, could block the cellular entry of VOCs, with noticeable inhibition rates [11]. In addition, sotrovimab appears to maintain its activity against all VOCs [12]. S1 receptor-binding domain (RBD)-targeted therapy, endosomal formation prevention and VOCs genome interruption have been proposed as potential treatment options against the new SARS-CoV-2 VOCs. However, recent findings regarding the use of monoclonal antibodies, convalescent serum and vaccines have raised concerns about using these agents for future variants [13]. These have prompted scientists to look for other safe and secure treatment strategies for COVID-19.

‘Herd immunity’ to COVID-19 has recently become a highly debated topic. When a large portion of a community develops ‘natural immunity’ to an infectious disease the term ‘herd immunity’ is used, however, achieving herd immunity for 50–66% of the population takes a long time, may result in many avoidable deaths and causes irreversible damage to the health system [13–15]. In contrast, immune-modulatory drugs and vaccination quickly achieve protective immunity against the infectious agent without devastating side effects. Accordingly, positive steps were taken by some companies to manufacture effective vaccines, some of which are now licensed for emergency use.

Although there is no effective treatment to combat SARS-CoV-2 infection, many efforts were made to reuse existing therapeutic agents, particularly anti-parasitic ones. Recent findings suggest that the anthelmintic agent albendazole appears to have a protective effect in COVID-19 patients with hydatid cysts caused by the tapeworm *Echinococcus granolusus* [16]. These intriguing results prompted us to review a few anti-parasitic drugs to evaluate their effectiveness against SARS-CoV-2. In addition, at the end of this review, we add brief descriptions about the application of new technologies such as artificial intelligence (AI) for developing new effective therapies against COVID-19. As far as we know, this is the first comprehensive review article that exclusively addresses anti-parasitic agents as potential therapies against COVID-19.

## Genome organization of SARS-CoV-2

The genomic sequences of SARS-CoV-2 and two bat coronaviruses, SLCoVZC45 and SL-CoVZXC21 (89–96.3% similarity), as well as human SARS-CoV (79–82%), are strikingly similar [17]. SARS-CoV-2 genome consists of 30 kb RNA that is protected at both ends by unique structures, including a 5’-cap and a 3’poly-A tail [18]. The novel coronavirus genome contains 14 open-reading frames (ORFs), which encode structural and nonstructural proteins. Out of them, 16 nonstructural proteins (nsp1–nsp16) are organized into two gene loci called ORF1a and ORF1b, which comprise 67% of the genome. They are located at the 5’ end of the genome and collectively mediate virus replication and possibly immune system evasion [19,20]. Furthermore, structural and accessory proteins are encoded by the remaining ORFs, which are located at the 3’ terminus of the genome [21]. There are four main structural proteins, including spike glycoprotein (S), envelope protein (E), membrane protein (M) and nucleocapsid protein (N). Two main subunits of spike surface glycoprotein, S1 and S2, are involved in the fusion of viral and host cellular membranes [22]. Therefore, a few studies indicated that the mutations in SARS-CoV-2 genome that include *S*, *nsp-1*, *nsp-3* and *nsp-15* may affect the virus’s interaction with the host [23,24].

## COVID-19 potential therapeutic strategies

To control the COVID-19 pandemic, considerable efforts were made to find suitable drugs [25]. Based on the virus’s life cycle, which includes assembly, budding and envelope formation, as well as pathogenesis [26], potential therapeutic strategies can be classified into the following categories: Blocking host–virus interaction using monoclonal antibodies or chemical agents that inhibit the virus from binding to host receptors [27], Blocking virus entry to host cells through inhibition of the clathrin-mediated endocytosis process [28–30], Neutralizing viral particles by disrupting viral enzyme activity and critical functional proteins involved in viral replication and multiplication [31–33] and Targeting viral structural proteins, including membrane, envelope and nucleocapsid proteins [34–36].

**Figure 1.**
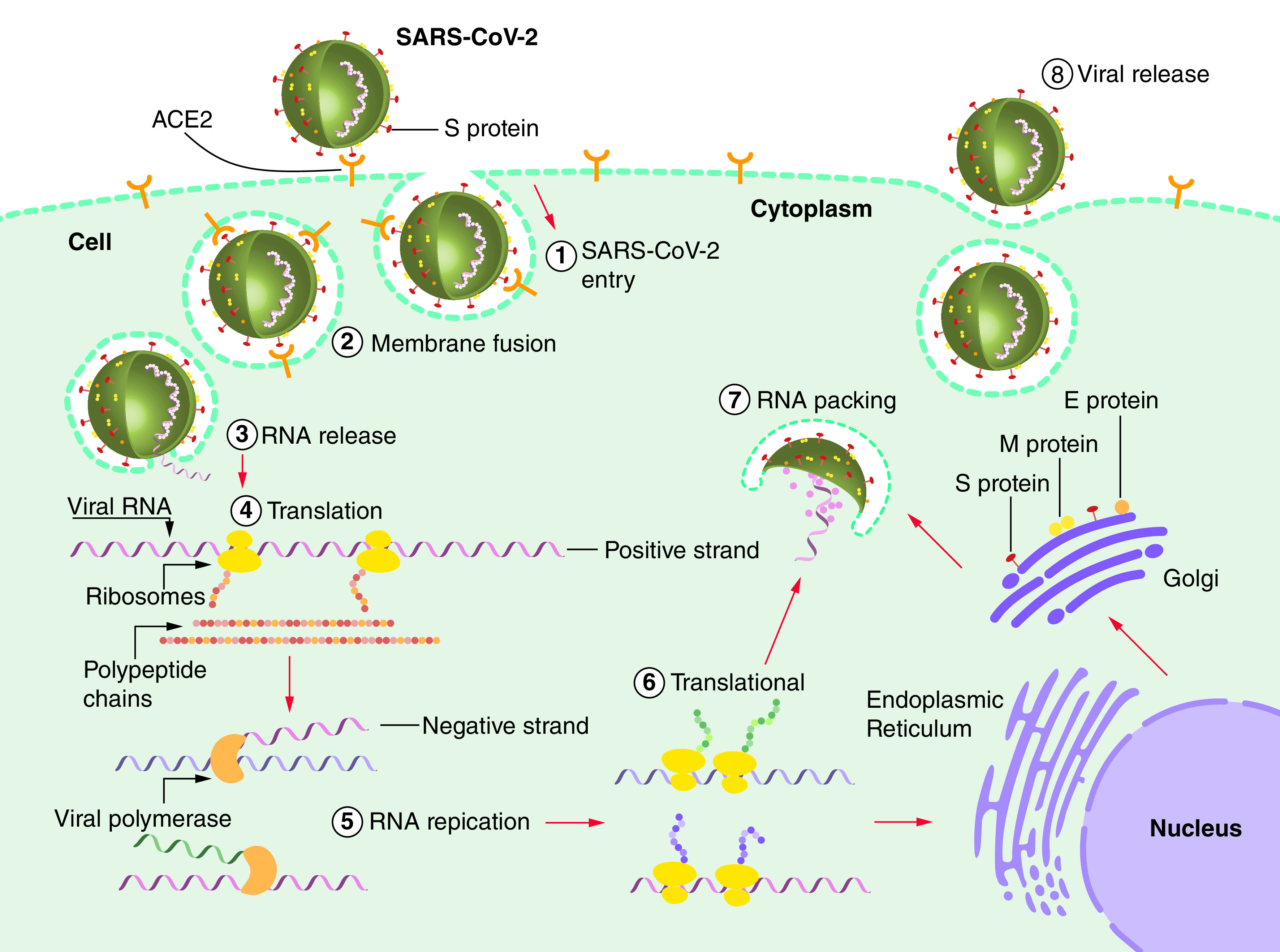
General mechanism of virus entry into the target cell. The RBD in the S1 subunit of the virus surface facilitates SARS-CoV-2 entry into host cells through the ACE2 receptor. The S2 subunit is involved in the fusion of the virus and target cell membranes and the following delivery of viral RNA into the cytoplasm. Following the entry and release of the viral genome into the host cell, the translated nonstructural polypeptides from two large ORFs form replication and transcription machinery to produce new virions. In the next step, translated structural proteins, including Spike, Membrane and Envelope, translocate to the ER and then from ER to Golgi intermediate complex so-called the ERGIC. Finally, encapsidated genomic RNA by Nucleocapsid assembles with structural proteins and is ready to release from infected cells through exocytosis. ER: Endoplasmic reticulum; ERGIC: Endoplasmic reticulum-Golgi intermediate compartment; E: Envelope; M: Membrane; N: Nucleocapsid; ORF: Open-reading frame; RBD: Receptor-binding domain; SARS-CoV-2: Severe acute respiratory syndrome coronavirus-2; S: Spike.

**Figure 2.**
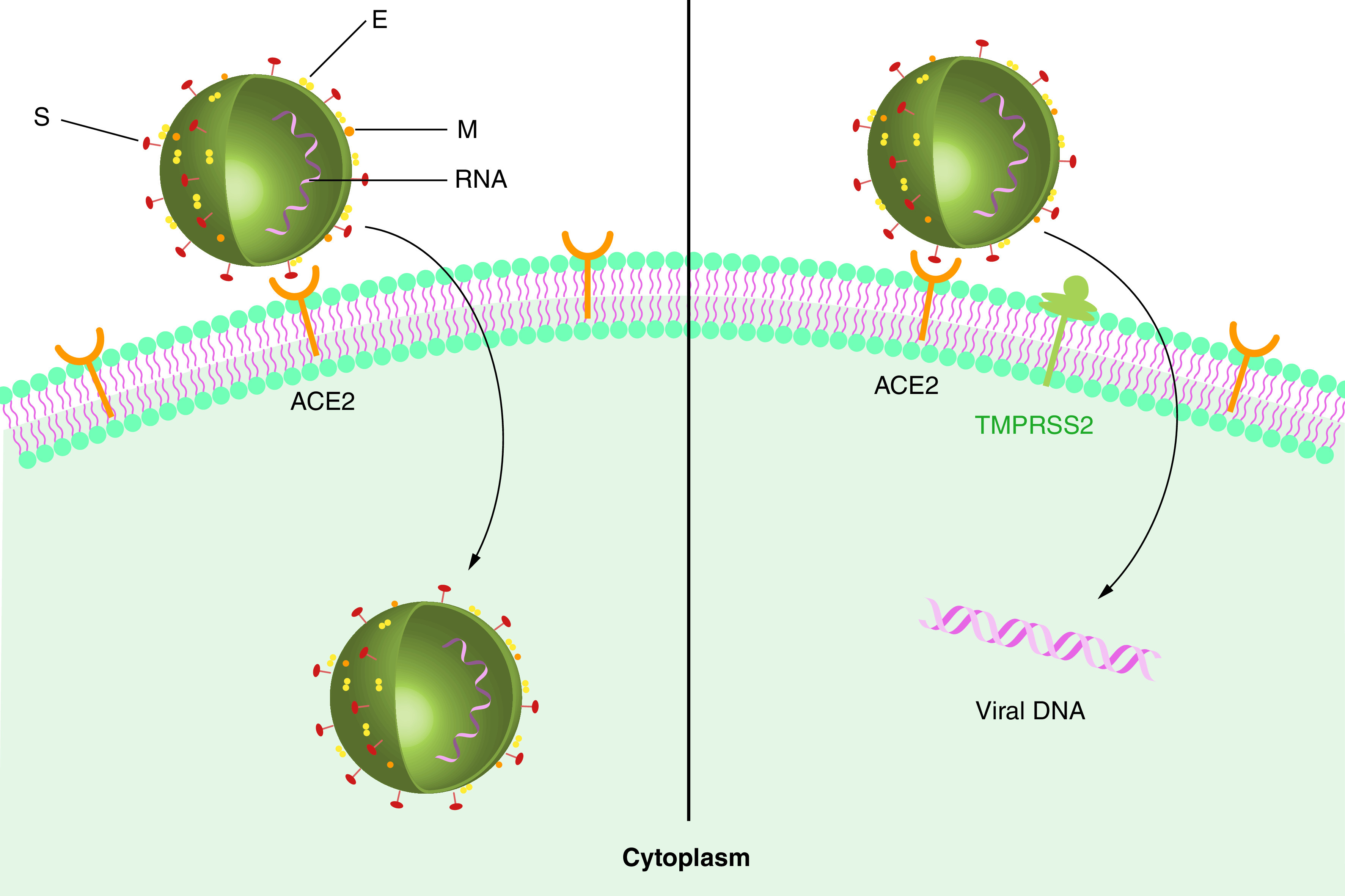
General mechanism of SARS-CoV-2 treatment: the role of type 2 transmembrane serine proteases. The TMPRSS2 found on host cells are involved in facilitating the viral entry into host cells, and TMPRSS2 inhibitors can significantly reduce infection. E: Envelope protein; M: Membrane protein; S: Spike protein; TMPRSS2: Type 2 transmembrane serine protease.

The first step of virus entry to host cells is the binding of the RBD of the viral S protein (S1) to angiotensin-converting enzyme 2 (ACE2) receptors (Figure 1), which is abundant in humans [4]. The viral spike glycoproteins are activated when they are cleaved by a serine protease. This facilitates virus–host cell membrane fusion necessary for virus entry, replication and distribution. Virus entry depends on the host’s trans-membrane serine protease, TMPRSS2 [37], which is proposed as a potential target for antiviral drug design. Therefore, the interaction between ACE2 and S protein can be targeted as an effective treatment strategy to combat the novel coronavirus [38]. In addition to the ACE2 receptor, host cellular serine protease is involved in facilitating the viral entry to host cells [39]; hence, TMPRSS2 inhibitors can significantly reduce infection as shown in cell lines with human lung origin (second strategy; Figure 2) [40–42]. A third strategy, potential antiviral drugs such as remdesivir can inhibit viral RNA-dependent RNA polymerase enzyme by being incorporated into the nascent viral RNA strands, which in turn causes premature transcription termination [43,44]. A fourth strategy that may be overlooked is to disrupt cellular packaging by interfering with the structural proteins of the virus. Among these proteins, N protein binds to the viral genome through its N-terminal and forms a ribonucleoprotein complex which has a principal role in viral replication and transcription [45]. Therefore, developing a potential drug candidate targeting this cross-linking prevents attachment of the N-terminal to viral RNA and may halt the viral replication and transcription [4,46]. It is well established that the structural proteins suppressing the host’s immune system play a central role in organizing the coronavirus assembly [47]. Moreover, the S protein, which is assembled on the surface of the viral particle, mediates binding and fusion to host cells and facilitates virus entry into the host cells [48]. Hence, M and S proteins can be potential therapeutic targets. E protein is another structural protein containing 76–109 amino acid and is essential in different stages of the virus life cycle such as envelope formation, pathogenesis, budding and virus assembly. Some studies have recently reported that E protein depletion results in an attenuated viral particle, which supports the idea that E protein may also be one of the potential therapeutic targets [49].

## Repurposed drugs to harness SARS-CoV-2

Only few countries were able to control the SARS-CoV-2 outbreak to some extent. Therefore, there is an urgent need to find new medications to protect humans against this disastrous pandemic. Given the urgency, repurposing existing the US FDA-approved medications is the most effective option for combating SARS-CoV-2. In this regard, some anti-parasitic agents have been suggested for the treatment of COVID-19 due to their potential effects in inhibiting SARS-CoV-2 [50] through inhibiting viral protease [51]. For instance, ivermectin (IVM) is an anti-parasitic agent that can reduce SARS-CoV-2 RNA load 5000-fold in 48 h post-infection *in vitro* [52]. Such reduction can potentially point out to the efficacy of IVM in treating COVID-19 with adequate dosing.

One of the most common clinical problems with the concomitant use of drugs is the problems caused by drug–drug interactions. For instance, coadministration of chloroquine (CQ) and paracetamol enhances the maximum serum concentration of paracetamol, which can cause side effects such as heart attack, stomach bleeding and kidney failure and so on. Also, concomitant administration of CQ and antacids causes a reduction in the absorption rate of CQ [53]. Furthermore, a growing body of evidence indicates that CQ and hydroxychloroquine (HCQ) coadministration cause QT-prolongation and arrhythmias. Although combination therapy is more effective than monotherapy, caution must be exercised in the concomitant use of these agents with antiviral drugs such as iopinavir/ritonavir, atazanavir, remdesivir and azithromycin (AZT) due to the increased risk of cardiac death [54].

Furthermore, according to the FDA fact sheet report, HCQ and CQ concomitant administration with remdesivir reduces antiviral activity of remdesivir [55]. This may be attributed to these agents being metabolized by the same cytochrome P450 (CYPs) isoenzymes. Studies have shown that IL-6, a proinflammatory cytokine increased in COVID-19 patients, inhibits the expression and activity of hepatic CYP isoenzymes, resulting in increased levels of drugs such as HCQ, CQ and remdesivir [56].

In the following sections, two main anti-parasitic drug categories are described, antimalarial and anthelmintic drugs, which are proposed as possible therapeutic options for SARS-CoV-2.

## Antimalarial drugs

Currently approved antimalarial medications apply two strategies to control SARS-CoV-2 outbreaks: those that aim to reduce the symptoms of the disease and those that prevent viral replication [57]. The potential antimalarial drugs against COVID-19 infection included CQ, HCQ, artesunate (ART), artefenomel (OZ439) and atovaquone (AV) [58].

### Chloroquine & hydroxychloroquine

**Figure 3.**
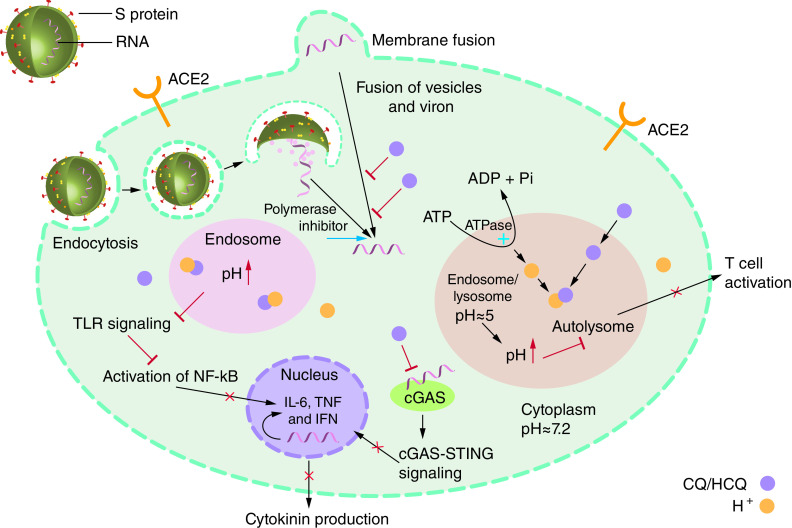
Possible mechanism of action of chloroquine and hydroxychloroquine in the intracellular space. In COVID-19, CQ and HCQ impede the virus entry into the cells by interfering in ACE2 glycosylation and decreasing ACE2 affinity for the coronavirus S protein. Furthermore, CQ/HCQ is known to interfere with the TLR pathway involved in proinflammatory cytokine signaling. Inhibition of endosomal/lysosomal acidification by preventing pH reduction to prevent the release of the viral genome into the host cell cytoplasm is suggested to be another mechanism. ACE2: Angiotensin-converting enzyme 2; cGAS: Cyclic GMP–AMP synthase; CQ: Chloroquine; HCQ: Hydroxychloroquine; S: Spike; STING: Stimulator of interferon genes; TLR: Toll-like receptor.

CQ and HCQ are quinine analogs that are derived from the *Cinchona officinalis* tree. HCQ is derived from CQ by introducing a hydroxyl group at the end of the side chain and was found to have better therapeutic effects on malaria due to a better safety profile, longer half-life, high levels of accumulation in cells and lesser drug–drug interactions [59]. They belong to 4-aminoquinolines class of medications that inhibit DNA and RNA polymerase have been used to treat malaria for the last 70 years [60]. Apart from malaria, CQ and HCQ are currently used to treat several viral infections in humans, as well as autoimmune diseases like systemic lupus erythematosus and rheumatoid arthritis [57,61]. These agents were proposed as a potential treatment for the novel coronavirus. CQ and HCQ share very similar structures and mechanisms of action, so both drugs may be useful to treat SARS-CoV-2 infection based on several *in vitro* studies [62,63]. Several modes of action were proposed to explain the therapeutic effects of CQ/HCQ, however, a precise underlying mechanism remains unknown. One possible mechanism is the inhibition of endosomal/lysosomal acidification by preventing pH reduction to prevent the release of the viral genome into the host cell cytoplasm [64]. In COVID-19, it is speculated that CQ and HCQ impede the virus entry into the cells by interfering in ACE2 glycosylation and give rise to decrease ACE2 affinity for the coronavirus S protein [65]. In the confirmation of this notion, the study on ACE2 high-expressed HEK293T cells (ACE2h cells) showed that the entrance of COVID-19 spike pseudotyped virus into ACE2h cells was suppressed by CQ and HCQ. Their results also showed that HCQ is slightly more toxic to ACE2h cells than CQ [66]. Furthermore, CQ/HCQ are known to interfere with the Toll-like receptor (TLR) pathway involved in proinflammatory cytokine signaling [67]; therefore, they suppress the immune system activation by down-regulating cytokine production and TLR–ligand interaction inhibition [68]. Both drugs can indirectly inhibit the production of IL-1, IL-6, TNFα, IFNα, MIP1β and IFNγ by various cell types with RNA-containing immune complexes. Hence, both drugs can interfere with antigen processing for MHC-II presentation through antigen-processing cells [69]. The detailed mechanism of CQ/HCQ action is presented in Figure 3.

CQ/HCQ safety was determined in malaria and individuals with autoimmune disease but not in COVID-19 patients [70]. In patients with COVID-19, especially those who have comorbidities such as endocrine disorders or cardiovascular diseases, CQ and HCQ use was reported to be challenging [71]. There are several studies, *in vitro* and *in vivo*, that show CQ and HCQ which affect various viruses, including influenza, HIV as well as SARS coronaviruses. In SARS coronaviruses their effects are attributed to impaired ACE2 receptor glycosylation [72–76]. In an *in vitro* study by Hu *et al.* for the evaluation of cytotoxicity and anti-SARS-CoV-2 effects of CQ and HCQ using Vero E6 cells 273.20 and 249.50 μm were indicated to be cytotoxic concentration, respectively. Thus, the dose-response curves of both drugs showed a noticeable effect on SARS-CoV-2, although the antivirus activity of CQ was higher than that of HCQ. Moreover, they were shown to inhibit the entry of the virus into host cells [72]. A similar study using the same cell line by Wang *et al.* showed the inhibitory effects of CQ alone or in combination with remdesivir; although the CQ itself was more effective than its combined form to control COVID-19 infection *in vitro* [75]. Using *in vitro* data and analysis by a specific computational method, Yao *et al.* conducted a study to determine the optimal dose of HCQ and CQ to use in the clinic. They found that HCQ has a more potent effect compared with CQ (EC_50_ = 0.72 vs 5.47 μm) on SARS-CoV-2 infection *in vitro*. Based on their analysis, a 400-mg HCQ and 200-mg CQ twice daily for 4 days is the optimal range [76].

Unlike *in vitro* studies, several clinical trials or observational studies have recently reported contradictory results regarding the safety and efficacy of CQ and HCQ in COVID-19 patients (see Table 1). For instance, a multicenter prospective study conducted by Huang *et al.* in China on 197 COVID-19 patients older than 18 showed no serious side effects in a full dose of CQ, while the patients who were treated with a half dose of CQ experienced a lower rate of adverse effects [77]. In contrast, a comparative study carried out in Brazil indicated a higher lethality and fatality rate for higher CQ dosages in a randomized, double-blind, parallel-group [78].

**Table 1.** Studies on some anti-parasitic drugs (chloroquine, hydroxychloroquine, ivermectin and nitazoxanide) in COVID-19 treatment.

Country	Date	Drug	Cell line or treatment	Sample size	Main findings	Ref.
*In vitro* studies						
China	February 2020	CQ and remdesivir	Vero E6 cells	Triplicates/different doses MOI	EC_50_ = 1.13 μm and CC_50_ >100 μm, High inhibition at low concentration	[75]
China	March 2020	CQ and HCQ	Vero E6 cells	Triplicates/different doses	CQ more potent than HCQ	[76]
China	July 2020	CQ and HCQ	HEK293T cells, HSAEpC cells, AT2 cells and EOL-1 cells	Both drugs at concentration of (0–400 μm), the toxicity and autophagy effects of drugs were evaluated on ACE2 high-expressed HEK293T cells	The entrance of COVID-19 spike pseudotype virus into ACE2h cells was suppressed by both CQ and HCQ	[66]
China	August 2020	CQ and HCQ	Vero E6 cells	Vero cells were infected at a multiplicity of infection of 0.01 (100 plaque-forming units/well)	HCQ more potent than CQ full-time entry, as well as postentry steps were inhibited by CQ and remdesivir	[72]
Germany	October 2020	Artemisinin-based treatments	VeroE6 and Huh7.5 cells	Vero cells were incubated in the presence of tenfold serial dilutions of the artemisinin derivatives for 15, 30, 60 or 120 min, before the virus was added at a concentration of 200 PFU per well for 120 min	Artesunate was the most effective inhibitor on SARS-COV-2	[131]
Australia	March 2020	IVM	Vero-hSLAM cells	Cells were seeded into 12-well tissue culture plates 24 h prior to infection with SARS-COV-2	Approximately 5000-fold reduction in viral RNA at 48 h	[52]

Clinical trials						
Brazil	January 2020	CQ	High dose: 600 mg CQ/BD/10 days or total dose 12 g; low dose: 450 mg/5 days, twice daily only on the first day or total dose 2.7 g	440 patients	High lethality and fatality at the higher dosage	[78]
China	February 2020	HCQ	1200 mg/day × 3 days then followed by a maintained dose of 800 mg/day daily/2 or 3 weeks	150 patients	Higher adverse events in HCQ recipients	[83]
China	February 2020	HCQ	400 mg/day up to 5 days	62 patients	HCQ could significantly reduce TTCR and promote the absorption of pneumonia	[84]
China	February 2020	CQ	CQ 500 mg twice daily up to 10 days + SOC	100 patients	Apparent efficacy and acceptable safety of CQ	[87]
China	March 2020	HCQ	400 mg/day/5 days	30 patients	No significant difference of the cure rate in case and control groups	[81]
France	March 2020	HCQ, AZT	600 mg/day/10 days	36 patients	12 cases were treated, enhanced effects in combination with AZT	[91]
France	March 2020	HCQ, AZT	HCQ 600 mg/day/10 days AZ 500 mg/day 1 and 250 mg/days 2–5	11 patients	No evidence of efficacy of the combination of HCQ and AZT for the treatment of hospitalized patients	[92]
USA	March–April 2020	HCQ	Exposure was defined as a prescription written for the drug as found in the electronic health record	1274 outpatients	Exposure to HCQ decreased the rate of hospitalization from COVID-19 outpatients (OR: 0.53; 95% CI: 0.29, 0.95)	[107]
Spain	March–April 2020	HCQ	Case arm: HCQ 800 mg/day 1, then 400 mg/day for 6 days Control arm: receive no specific therapy	Case arm: 1116 patients control arm: 1198 patients	No positive effect of postexposure therapy	[98]
Pakistan	April 2020	HCQ	Case group: HCQ 400 mg/BD/day 1, then 200 mg/BD/4 days Control group: SOC (Vit C, Vit D and zinc only)	500 patients with mild COVID-19 (control group: 349 patients received SOC, case group: 151 received HCQ)	HCQ combined with SOC in mild COVID-19 neither prevents disease progression nor is it significantly associated with successive PCR negativity on day 7	[100]
France	April 2020	CQ, AZT	HCQ 200 mg three-times/day/10 days AZT 500 mg/day 1 followed by 250 mg/day up to 4 days	80 patients	HCQ may be in force in lowering respiratory viral load and patient carrying duration, augmented by AZT	[90]
France	May 2020	HCQ	600 mg/day/21 days	Treatment group: 84 Control group: 89	Do not support drug use in hospitalized patients	[93]
USA	May 2020	HCQ, AZT	HCQ 600 mg/BD/day 1, then 400 mg/day/4 days	1376 patients	HCQ use was not related to noticeable lower or higher hazard of death or intubation	[94]
China	May 2020	HCQ	200 mg/BD/7–10 days	Case group: group: 48 Control group: 520	The mortality and inflammatory cytokine IL-6 decreased in case group	[85]
USA	May 2020	HCQ, AZT	HCQ 200–600 mg AZT 200–500 mg once alone or at combination	1438 patients	Treatment with HCQ, AZT alone or both was not decreased the mortality of hospitalized patients	[95]
Korea	June 2020	HCQ	400 mg/day/14 days	189 patients	Safety results in postexposure prophylaxis	[80]
England	June 2020	HCQ	800 mg/BD/day 1, then 400 mg/BD/9 days	1561	Severity and comorbidities	[101]
China	September 2020	CQ	500 mg once (half dose) or twice (full dose) daily	197	No adverse side effects, especially in half dose	[79]
China	February 2020	CQ, HCQ		CQ = 40, HCQ = 40 Control group = 20	Phase 0	ChiCTR2000030054
Egypt	March 2020	CQ, HCQ		200 participants	Phase II, III	NCT04353336
England	April 2020	CQ, HCQ		40,000 participants	Phase not applicable	NCT04303507
Egypt	June 2020	CQ, HCQ vs remedesivir		120 patients	Phase II, III	NCT04345419
Bangladesh	June 2020	IVM-doxycycline and HQC-AZT	Group A: IVM 200 μg/kg single dose + doxycycline 100 mg BD for 10 days Group B: HQC 400 mg first day, then 200 mg BD for 9 days + AZT 500 mg	Group A: 60 patients Group B: 56 patients	IVM-doxycycline had better effect on reducing symptoms, recovery duration time and adverse effects compared with group B	NCT04434144 [86]
USA	August 2020	IVM	IVM at 200 μg/kg repeated at day 7	Unmatched cohort: 280 patients Matched cohort: 196	IVM therapy is associated with lower mortality, especially in patients needed oxygen and ventilatory support	[152]
Iraq	October 2020	IVM-doxycycline	IVM at 200 μg/kg/day/2–3 days + 100 mg doxycycline/BD/5–10 days	70 COVID-19 patients (48 mild–moderate, 11 severe and 11 critical patients)	Reducing recovery time and progress to more advanced stage; Significantly reducing mortality rate in severe patients	[154]
Egypt	June 2020	IVM	IVM: 3 successive days/three-times a day, started within 48 h of symptoms	100 participants	Phase II, III	[NCT04445311]
Colombia	October 2020	IVM	IVM 400 μg/kg (2 drops per kg) orally in a single dose	50 patients	Phase II	[NCT04602507]
Iraq	April 2020	IVM, HCQ AZT	IVM: Single dose 0.2 mg/kg HCQ: 400 mg BD in first day then 200 mg BD/5 days AZT: 500 mg in first day then 250 mg/5 days	16 participants	Phase I	[NCT04343092]
Argentina	May 2020	IVM	IVM: 600 μg/kg once daily plus standard care	45 participants	Phase II	[NCT04381884]
USA	May 2020	IVM	Days 1–2: weight <75 kg: four tabs (12 mg total daily dose) Days 1–2: weight >75 kg: five tabs (15 mg total daily dose)	240 participants	Phase II	[NCT04374019]
Bangladesh	August 2020	IVM, doxycycline	IVM: 6 mg Doxycycline: 100 mg BD/5 days	400 participants	Phase III	[NCT04523831]
Colombia	March 2021	IVM		400 patients 300 μg/kg of body weight per day for 5 days (n = 200) or placebo (n = 200)	Did not improve the time to resolution of symptoms Do not support the use of IVM for mild COVID-19	[155]
Bangladesh	February 2021	IVM, IVM-doxycycline		Oral IVM alone 12 mg once daily for 5 days 12 mg IVM single dose and 200 mg doxycycline on day 1, followed by 100 mg every 12 h for the next 4 days	Earlier virological clearance in IVM group	[157]
Brazil	December 2020	NTZ, Placebo	NTZ or placebo: 500 mg/3-times/5 days	392 patients	Symptom resolution did not differ between two groups, significantly reduced viral load	[169]
Egypt	April 2020	IVM plus NTZ	IVM: 200 μg/kg once + NTZ: 500 mg/BD/6 days	100 participants	Phase II/III	[NCT04360356]
Egypt	May 2020	NTZ	–	160 participants	Phase III	[NCT04382846]
Egypt	February 2021	NTZ–ribavirin-IV-zinc	NTZ500 mg rapid release formula/6 h ribavirin 1200 mg (400 mg divided doses) IVM weight dependent zinc 30 mg twice daily	113 patients	Decreasing the duration of viral nasopharyngeal clearance	[170]
Brazil	July 2021	AZT plus NTZ, IVM or HQC	AZT 500 mg daily for 5 days for all patients, in association with one of the following: HCQ 400 mg daily for 5 days, NTZ 500 mg twice a day for 6 days, or IVM 0.2 mg/kg/day in a single daily dose for 3 days	722 patients	There were no actual effective options for early COVID-19, in other words, that none of the drugs would confer any protection	[175]
Nigeria	6 October 2020	NTZ, atazanvir, aitonavir	1000 mg of NTZ twice daily orally and 300/100 mg of atazanvir/ritonavir once daily orally	98 Patients	Phase II	[176]
Egypt	June–July 2020	AZT, doxycycline, NTZ	AZT was 500 mg once daily for 5 days; doxycycline 100 mg daily for 10 days; NTZ was 600 mg twice daily for 5 days	80 patients	Symptomatic improvement of mild-to-moderate subjects was seen on the fifth or seventh day after starting treatment. Both NTZ and doxycycline have great therapeutic potential against COVID-19	[177]
Mexico	1 May and 20 July 2020	NTZ	NTZ 500 mg orally, every 6 h for 2 days and then 500 mg twice a day for 4 days	150 Patients	NTZ prove to be useful against SARS-CoV-2 as an early intervention to avoid complications	[178]
Argentina	July–December 2020	NTZ	Patients receive NTZ orally with food for 14 days	46 patients	The ratio of patients with a viral load reduction ≥35% from baseline up to day 7 of treatment was significantly greater for NTZ compared with placebo	[179]

AZT: Azithromycin; CQ: Chloroquine; HCQ: Hydroxychloroquine; IVM: Ivermectin; NTZ: Nitazoxanide; OR: Odds ratio; SOC: Standard of care; TTCR: Time to clinical recovery; Vit: Vitamin.

In addition, the prophylactic or postexposure prophylactic administration of CQ and HCQ has not shown any efficacy in COVID-19 patients [79,80]. Furthermore, a systematic review analysis indicated that treatment of hospitalized COVID-19 with CQ/HCQ might not reduce the risk of death and infection rate [81,82]. Another recent systematic review illustrated no obvious profit in CQ/HCQ use for the treatment of COVID-19 or prophylaxis against this disease [83].

As a CQ derivative, HCQ is believed to be effective in mild-to-moderate cases of SARS-CoV-2 patients when used as monotherapy or in combination with CQ or AZT. In this regard, Chen *et al.* conducted a randomized clinical trial study to evaluate HCQ efficacy in 30 adult COVID-19 patients in comparison to a control group including patients with liver abnormalities, anemia and renal dysfunction. Although the prognosis of these COVID-19 patients was good in this small pilot study, the symptom improvement and cure rate in the COVID-19 group did not differ from the control group [84]. In another study, mortality rate and inflammatory cytokine IL-6 levels decreased versus HCQ-treated group [85]. In a recent trial conducted on mild-to-moderately ill patients without comorbidities or with near-normal chest radiographs, HCQ-AZT combination therapy was able to reverse PCR results within 7 days and abolish all symptoms by 9 days [86]. Another clinical trial study by Gao *et al.* was conducted to evaluate the efficacy and safety of CQ phosphate in 100 hospitalized COVID-19 patients who also suffered from pneumonia. Following the treatment, the severity of pneumonia was decreased in patients without any severe adverse reactions. Given these results, the authors recommended using this drug for the prevention and treatment of pneumonia caused by COVID-19 [87,88].

In an randomized clinical trial (RCT) study, a randomized and parallel control group study in Renmin Hospital of Wuhan University, China, the efficacy of HCQ was evaluated in 62 COVID-19 patients, which resulted in a shortening of the recovery time in the patients who received HCQ [89]. Although there are some concerns about the arrhythmogenic (torsadogenic) effect of CQ/HCQ alone, it was shown that coadministration of AZT with CQ reduces this effect [90]. A small-scale nonrandomized open-label trial study investigated HCQ effect alone or combined with AZT on respiratory system viral load. The results showed that HCQ combined with AZT versus HCQ alone was able to treat 100% of patients [91]. Unfortunately, despite the reduction in viral load, the authors overlooked the assessment of QT prolongation when both drugs are coadministrated. Furthermore, a systematic review and meta-analysis study by Fiolet *et al.* [92] and others found that HCQ with AZT increased the mortality rate in hospitalized patients compared with HCQ only [93]. In observational study by Geleris *et al.*, they did find no association with either a greatly lowered or an increased risk of the composite end point of intubation or death [94]. In another research, investigators did also not find significant effect on mortality rates among patients hospitalized with COVID-19 treated with HCQ, AZT or both [95].

Continuing to investigate HCQ/CQ as a potential treatment for COVID-19, Hussein and Elkhair conducted a molecular docking survey to improve CQ and HCQ efficiency. Using molecular docking and molecular dynamics methodologies, they showed that the addition of zinc compounds to CQ/HCQ enhances their activity as potential inhibitors of COVID-19 main protease [96].

Despite positive results of some studies, some others stood in contrast and reported the opposite results regarding CQ/HCQ efficacy to treat COVID-19 patients [97,98]. In a multicenter study conducted in France, following administration of HCQ (600 mg/day), it was reported that administration of HCQ had no effect in COVID-19 patients who were admitted to the hospital [99]. Moreover, in a randomized trial involving 491 outpatients with mild disease in early stages, HCQ was not effective in reducing symptom severity [100]. Another large-scale randomized trial involving more than 4000 hospitalized patients showed no difference between patients who received HCQ and those patients undergoing usual care at 28 days in death incidence [101].

Hypothetically, HCQ can fight SARS-CoV-2 infection due to its mode of action. HCQ interferes with viral entry into host cells [102]. Based on another hypothesis, HCQ can prevent SARS-CoV-2 infection in healthy persons who are exposed to PCR-positive patients. Mitja *et al.* conducted a large-scale open-label cluster-randomized clinical trial in Spain to test this hypothesis. After analysis, they concluded that postexposure therapy with HCQ does not prevent infection in healthy contacts [103]. A similar study to test this hypothesis was conducted on participants who had a high risk of exposure to COVID-19 in a house or occupational office. Similar to the previous study, they did not find any preventive effects of HCQ [104]. A study designed by Xie *et al.* to evaluate the efficacy and safety of HCQ in 150 hospitalized adult patients with COVID-19 showed higher adverse events in HCQ plus standard-of-care (SOC) recipients than in HCQ nonrecipients. The authors concluded no significant difference between HCQ and SOC groups in conversion of mild-to-moderate condition [105]. In agreement with the aforementioned results, another study confirmed that the addition of HCQ to SOC in mild COVID-19 cases neither stops disease progression nor helps with early and sustained viral clearance [106].

However, the results of a recent study conducted by Ip *et al.* showed that although HCQ has not been associated with improved survival among hospitalized COVID-19 patients, there is an association between HCQ administration and a decreased rate of hospitalization of COVID-19 patients who are mildly symptomatic. According to their results, the subsequent hospitalization rate was declined with HCQ exposure [107].

All clinical trials related to CQ/HCQ presented here have been collected from the clinicaltrials.gov database until 20 May 2021 (https://clinicaltrials.gov/ct2/results/details?cond=COVID-19). The database search resulted in 89 and 22 completed clinical trials for HCQ and CQ, respectively. During this period, 180 and 40 clinical trials have been registered that have not yet been completed. Some of them demonstrated good virological and clinical outcomes with HCQ and CQ alone or in combination with other anti-COVID-19 agents. Nevertheless, most trials had varying degrees of methodological limitations. In contrast, some studies either showed a negative result or did not show any changes with CQ/HCQ exposure compared with the control groups. So far, numerous studies have reported serious adverse effects caused by CQ and HCQ, although uncommon [108–110]. These include hemolysis [111], cardiac toxicity in the form of cardiomyopathy [109], and prolonged QTc interval [110,112]. Van den Broek *et al.* studied the degree of CQ-induced QTc interval prolongation in hospitalized COVID-19 patients. In a total of 95 patients who were suspected of COVID-19 infection, ECG was collected pre-/post-treatment with CQ. About 23% of patients showed a QTc interval of more than 500 ms during CQ therapy, which represents a significant role of CQ in prolonging QTc interval in a clinically relevant manner [110].

Based on the NIH panel, the combined use of HCQ plus AZT and high-dose CQ (600 mg twice daily for 10 days) due to the potential for toxicity has not been recommended [113]. Given these controversial results, the WHO removed these drugs from joint international trials because they were not effective versus SOC as recommended by steering committee’s recommendation [114].

In general, as mentioned above, different studies have got contradictory results considering CQ/HCQ efficacy in COVID-19 patients [115], which may be in part attributed to various study designs, sample sizes and various statistical procedures as well as population genetics. Therefore, it may be necessary to carry out clinical trials on a larger scale taking into account different populations, races, demographics and patient-related factors; for example, are the patient hospitalized or not [88,116]?

### Artesunate

ART, one of the semisynthetic derivatives of artemisinin, is a vital sesquiterpene lactone obtained from *Artemisia annua* leaves. As one of the biologically active compounds against malaria, and taking into consideration its low toxicity, rapid distribution, high efficiency, high solubility in water and how well it is tolerated, ART was proved to be a standard treatment for cerebral malaria and other severe forms of malaria [117]. In traditional Chinese medicine, it was used for the treatment of several diseases for more than 2000 years [118]. Furthermore, it is indicated that ART has broad antiviral activity against certain viruses, such as human cytomegalovirus, hepatitis B and C virus, herpes virus and bovine viral diarrhea virus [119,120]. ART was recommended by some investigators to be used for the treatment of patients infected with SARS-CoV-2; however, the exact molecular mechanisms by which ART can affect SARS-CoV-2 have not been elucidated. Antiviral properties of ART may be attributed to inhibition of transcription NF-κB expression and disrupting viral protein synthesis as well as to blocking the early steps of viral replication [120,121]. It is known that the NF-κB is the master regulator of host immune and inflammatory responses against invasive pathogens [122,123]. SARS-CoV-2, like other families of Coronaviridae, primarily targets the upper respiratory tract [124], so it causes excessive proinflammatory host responses that induce immune pathology and can harm lung tissue [125]. The idea that ART can affect COVID-19 comes from the fact that it can inhibit the production of IL-1B, IL-6 and IL-8 by inhibiting NF-κB translocation in a dose-dependent manner *in vitro* [126]. Elevated IL-6 serum levels in COVID-19 patients may be a sign of cytokine release syndrome, suggesting that controlling IL-6 could decrease the natural course of the disease [127]. Recent studies have shown that ART is active against SARS-CoV-2 [128–131]. For instance, Gilmore *et al.* used artemisinin and its synthetic derivative as inhibitors of SARS-CoV-2 *in vitro* [131]. Their results showed that ART is a more effective inhibitor of SARS-CoV-2 compared with *A. annua* extracts [131,132]. In another study, Cao *et al.* evaluated the anti-SARS-CoV-2 potential of nine artemisinin-related compounds *in vitro*. Their results highlight that the artemisinins could be considered potential anti-SARS-CoV-2 candidates in drug research and development [128,133,134]. In addition, positive clinical effects of artemisinin have been shown recently. The drug hampers the progression of the symptoms in mild/moderate form of COVID-19 [135].

### Atovaquone

Atovaquone, an analog of ubiquinone, is a highly lipophilic hydroxynaphthoquinone. It is used against *Plasmodium falciparum* and *Pneumocystis carinii*, which cause malaria and pneumonia, respectively. It selectively inhibits the parasite’s mitochondrial cytochrome bc1 complex (complex III) [136]. However, the underlying molecular mechanism against *P. carinii* has not been fully elucidated.

A clinical trial is underway by HonorHealth (AZ, USA) to answer the question of whether coadministration of atovaquone and AZT have more advantage in COVID-19 patients over other treatments? Their results showed fewer cardiac side effects; however, available data are insufficient to draw judgments about the effects of atovaquone as a COVID-19 treatment [137].

Although clinical trials on the function of atovaquone in COVID-19 patients are limited, however, some *in silico* studies have proposed that it may be suitable to control this pandemic.

In this regard, molecular docking analysis based on the docking score as well as its binding energy among 129 drugs showed that atovaquone could be suitable to control the novel coronavirus disease [138]. A similar study published in preprint server, which used structure-based drug modeling design, found similar results and demonstrated that atovaquone was among the top three candidates [139]. In contrast, an *in silico* survey among 13 approved antimalarial drugs gathered by docking analysis against two specific targets, spike antigen and main protease of the SARS-CoV-2, indicated that the atovaquone is one of the moderately effective drugs based on the g-score [58]. Another computational study showed a good connection between the IVM, atovaquone and some antimicrobial agents with SARS-CoV-2 protease enzymes [140]. More experimental research is needed to confirm the sensitivity and specificity of this agent [138,140].

Recently, a study showed the potential antiviral effect of atovaquone on emerging VOCs of SARS-CoV-2 *in vitro* by interfering in viral replication at the postentry phase. However, they concluded that there is a need to conduct additional clinical studies with using either atovaquone alone or in combination with other recommended drugs for COVID-19 [141].

## Antihelminthic drugs on COVID-19

### Ivermectin

**Figure 4.**
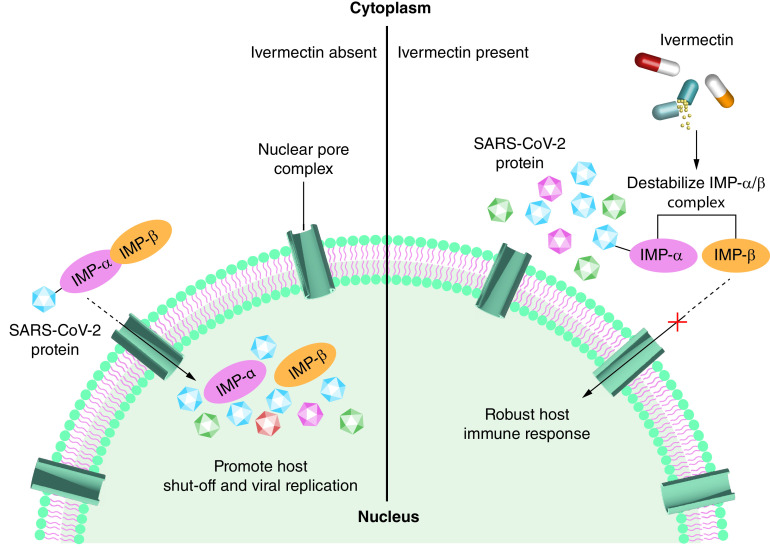
Mode of action of ivermectin in the intracellular space. IVM destabilizes the IMP-α/β complex and robust host immune response. In the absence of IVM, this complex enters the nucleus resulting in viral replication. IVM: Ivermectin; NPC: Nuclear pore complex.

IVM is frequently used as an antistrongyloidiasis, ascariasis and onchocerciasis drug. It was confirmed as an appropriate therapy for some other parasitic micro-organisms [142,143]. Apart from its conventional use, IVM clearly has shown anti-COVID-19 properties *in vitro*. It seems that its mode of action may be through inhibiting the translocation of viral components into the nucleoplasm, which is mediated by ‘importin α/β1’ (Figure 4) [52]. An *in silico* study conducted for decrypting the binding mode of IVM interaction with potential drug targets associated with COVID-19 showed that IVM interacts with a strong and moderate affinity to Nsp9 and IMPα, respectively [144]. By the same mechanism, IVM also inhibits replication of other RNA viruses like HIV, influenza, yellow fever, etc. [145–148]. Besides, it was suggested that the accumulation of two IVM molecules together onto the virus capsid causes the formation of an ionophore structure that osmotically causes the virus disintegration [149]. However, these hypotheses should be tested before using this agent as a therapeutic arm against COVID-19.

Despite the significant effects of IVM *in vitro* [52], due to its neurotoxic side effects at high concentrations in humans, its use for clinical trials requires careful risk–benefit considerations, especially in critically ill patients. However, the presence of higher levels of the drug in lung tissue than plasma after 1 week of oral administration has raised hopes to bypass its dose-dependent side effects [146]. In comparison to HCQ with side effects including retinal damage and elevated QT interval, IVM has fewer side effects [150]. As usual, several clinical trials are ongoing to test the potency of IVM as an anti-parasitic drug against COVID-19. For instance, in a randomized and comparative clinical study of IVM-doxycycline and HCQ-AZT therapy for COVID-19, findings showed that the combination of IVM-doxycycline had an even higher trend of superiority to the HCQ-AZT in low-risk patients. However, these differences are not statistically significant [86]. A randomized controlled trial study showed that in the early stages of the disease, IVM with doxycycline treatment has clearly prevented the progression of the disease to higher stages and significantly decreased mortality rates in severe patients [151]. It was suggested that the administration of IVM and HCQ together could have synergistic effects in COVID-19 patients [102]. HCQ fights the virus by preventing it from entering the host cell, and IVM combats the virus by inhibiting viral replication. However, basic and clinical studies are needed to reveal the possibility of this combination being used as an acceptable COVID-19 treatment [102]. Although IVM treatment of 173 hospitalized COVID-19 patients resulted in lower mortality in both severe and nonsevere groups with pulmonary involvement compared with nontreated groups [152]. However, this study and a similar study [145] carried out by the same team suggested that more studies, including randomized controlled trials, are needed to clarify the effect of this drugs on SARS-CoV-2 in comparison to control groups [152,153]. A randomized double-blind placebo-controlled small-scale trial also revealed that 5 days of treatment with IVM increased the virological clearance in hospitalized adult COVID-19 patients with mild disease [154]. In contrast, another similar study on adults with mild COVID-19 who were treated with a 5-day course of IVM showed that the time of symptom resolution was not affected significantly. They suggested that IVM is not a good treatment for mild cases [155] (for more details, see Table 1).

Nevertheless, a recent meta-analysis study revealed that IVM reduced the risk of death compared with no IVM [156]. Additionally, *in silico* study by using AI-based and classical simulation methods indicated positive interaction between IVM and viral protein targets; however, it requires evidence from clinical studies to support its use [157]. In confirming the significance of IVM, a web-based reported meta-analysis of 65 studies revealed that there is an statistically significant improvements for mortality, ventilation, ICU admission, hospitalization, recovery, cases and viral clearance by using IVM [158].

While in March 2021, the WHO recommended that IVM could be used in clinical trials, new reports indicate that COVID-19 patients may die from its poisoning. Therefore, larger clinical trials may be needed to uncover the efficiency of IVM in COVID-19 patients.

### Nitazoxanide

Nitazoxanide (NTZ), as a pyruvate-ferredoxin oxidoreductase inhibitor, inhibits a wide range of RNA and DNA virus replication, including influenza, parainfluenza, rotavirus, coronavirus and so on [159–162]. The antiviral activity of NTZ has already been demonstrated on MERS by inhibiting the production of nucleocapsid protein *in vitro* [163]. Recently, it has also been shown that it can prevent the SARS-CoV-2 growth in the vero cell model [75].

At least one study has suggested that dual therapy can be highly effective against SARS-CoV-2 by a multitude of cellular and molecular mechanisms. HCQ prevents the virus from entering the cell; on the other hand, NTZ increases the innate interferon-dependent immune responses [164]. Furthermore, NTZ promotes and upregulates other innate immune components, including MDA5, RIG-1, MAVS [165]. Moreover, due to its specific antiviral effects, NTZ may also have anti-inflammatory activities by reducing IL-6 and TNF-α. Considering hyperinflammation and cytokine storm caused by SARS-CoV-2, it may improve patient prognosis even though direct evidence is lacking [166,167]. It is well established that NTZ and niclosamide (NIC), two potent TMEM16A antagonists, possess bronchodilatory properties which may effectively improve respiratory symptoms, decrease shortness and tightness of breath, and facilitate respiration and pulmonary ventilation in COVID-19 patients [168]. Despite the several above-mentioned useful effects, unfortunately, due to existing confounding data in the literature, limited clinical trials have been ongoing to evaluate NTZ efficacy on COVID-19 patients (Table 1) [164]. For instance, in a double-blind randomized multicenter clinical trial, early-stage treatment of 194 COVID-19 patients by NTZ resulted in significantly reduced viral load but did not affect symptom resolution compared with the placebo group. This study also showed that neither were the disease markers significantly affected nor were significant side effects observed [169]. In contrast, a combination therapy including NTZ, ribavirin, IVM plus zinc resulted in significantly increased SARS-CoV-2 clearance from the nasopharynx compared with symptomatic treatment [170]. Another set of clinical trials is under design with NTZ monotherapy or its combination with other therapies, including HCQ or AZT [164,171].

According to the findings of a systematic review, NTZ is considered safe at the approved doses. However, further evidence is required for its cardiovascular, hepatorenal and teratogenic consequences [172]. Dose predictions were performed to achieve appropriate plasma and lung concentrations effective against SARS-CoV-2 according to its specific EC_90_, and adjustments were made for subsequent clinical trials [173]. Further studies are given in Table 1 [174–179].

### Niclosamide

NIC is an anthelmintic drug with anti-inflammatory and immune regulatory effects and acts by interfering with the oxidative phosphorylation pathway. Besides, it has recently been used as a potential anticancer, antibacterial and antiviral agent in addition to what has been stated [180]. Its inhibitory effects on Zika virus replication, an RNA virus responsible for infection of astrocytes and neural progenitor cells were identified [181]. Furthermore, NIC has previously been shown to have anti-SARS-CoV-2 effects by inhibiting viral replication through blocking virus spike and nucleocapsid antigens production [182,183]. It has also recently been shown that NIC has anti-SARS-CoV-2 and anti-MERS-COV properties *in vitro* [184]. In an experiment, two stable forms of NIC (C1 and C2) were assessed to determine their solvation energy and reactivity with COVID-19 proteins in different media (gas and aqueous media) compared with eleven other antivirals. C1 and C2 forms showed higher reactivity with COVID-19 proteins, despite their lower solvation energy which is attributed to the NO2 and Cl groups in the active site of their structures [185]. NIC also ranked at the top of the list of 1553 FDA-approved and 7012 off-market drugs since it has low binding energy and high affinity for COVID-19 protease. Possible other modes of action, including prevention of endocytosis and deactivation of S-phase kinase-associated protein 2 (SKP2), may contribute to NIC anti-SARS-CoV-2 activity [186,187].

Hypothetically SARS-CoV-2 infection declines autophagy; hence, the addition of autophagy-inducing compounds can be considered a treatment goal against COVID-19. Autophagy inhibition could increase the replication of the virus by stopping the segmentation of viral antigens. In this regard, an *in vitro* study suggested that NIC as an autophagic cell death inducer agent can be used as prophylactic treatment during SARS-CoV-2 infection [188]. Furthermore, molecular docking analysis indicated that the NIC and other antihelminth drugs such as primaquine, mepacrine, artemisinin could bind to the active site of the SARS-CoV-2 protease. This study has also shown that the type of interaction between ligands and virus protein is important in therapy [189]. A similar computational study confirmed that NIC could bind with SARS-CoV-2 protease with high affinity [185]. Recently, several clinical trials have been ongoing to test the efficacy of NIC alone or combined with HCQ against COVID-19 (Table 1). For instance, the intranasal and inhalation form of NIC was evaluated for their safety and possible side effects. The results indicated that this solution might serve as the primary eradicator of SARS-CoV-2 from the upper respiratory tract without adverse effects other than temporary mild stimulation [190].

### Mebendazole

As an anthelminthic drug, mebendazole (MBZ), along with other benzimidazoles, was found to have antiviral properties and to be efficient against certain viruses such as HSV-1, CVB-2 and Zika virus [181,191,192]. According to molecular docking analysis, MBZ with two other compounds, atovaquone and ouabain, showed anti-SARS-CoV-2 properties *in vitro* [193]. In a single-cell RNA sequencing dataset analysis obtained from mild-to-severe cases of COVID-19 patients, MBZ also ranked in the top ten compounds based on connectivity score. This study also indicated that the immune profile of patients in response to different viral infections impacts the efficacy of different compounds against those infections [194]. Based on the calculated binding energy and affinity, MBZ has also been ranked in the top 30 FDA-approved drugs that interfere with SARS-CoV-2 activity [186]. MBZ and similar structure drugs such as albendazole, and oxibendazole may act against COVID-19 by compromising cellular microtubule integrity, which interferes with the cellular trafficking of the virus [195]. It was shown that MBZ is one of the BCG mimics, which promotes innate immune responses and is effective against new infections like SARS-CoV-2 [196].

## AI, a state-of-the-art technique for finding new drugs

The emergence of the SARS-CoV-2 virus and COVID-19, the disease it causes, in the world has led to a widespread effort to find suitable treatments for this aggressive pathogen. Considering the urgency and rapid transmission, examining existing medications that are approved for human use will be the right strategy to combat the virus. Unfortunately, there is currently no certain effective treatment, and according to the recent solidarity study conducted by the WHO [194,197], compared with placebo, none of the retasked drugs, including remdesivir, HCQ and IFN-β as a single, as well as IFN-β plus lopinavir and lopinavir coadministered with ritonavir as a combinatorial therapy, were effective in survival, initiation of ventilation and hospitalization length in COVID-19 patients. Therefore, finding suitable candidates for new medications is still ongoing.

One of the most important translational research activities contributing to human well-being and health is drug discovery and design [198]. While the physicians’ resort to trial-and-error techniques in hospitals due to the inefficiency of the lab-based high-throughput screenings, virtual screening and molecular docking have emerged as a common tool to discover powerful compounds against SARS-CoV-2 [199–205]. In general, this computational approach applies chemically and biologically algorithms in large chemical libraries to find appropriate hits based on the known structure of the target or the ligands. Although this method has had a lot of success in recent years in discovering new drugs such as nelfinavir and zanamivir, it still faces various challenges such as sampling various conformations of flexible molecules and calculating binding energy between receptor and ligand [206], as well as simulation cost and exhaustive similarity searching [207]. With traditional methods, *in silico* HTS simulation performance is not simple because of intensive computational model calculations and taking an incredible running time [208].

We have recently come across an unprecedented wealth of data in the chemical and pharmacological fields that can feed state-of-the-art methods such as AI algorithms in drug discovery or design. Unlike traditional methods, this method does not rely on developing complicated physical and chemical concrete principles and only turns the large amount of medical data currently available into reusable knowledge [209]. In different phases of the drug development process, including target validation, assay development, HTS, hit to lead, lead optimization, preclinical and clinical development as well as drug repurposing, AI-based approaches are increasingly being used to boost time and cost-efficiency [198].

There is still no consensus definition for AI. In general, it is a branch of computer science whose main purpose is to produce intelligent machines capable of performing tasks that require human intelligence. AI applications cover a wide range of disciplines, including computer vision, voice recognition, language understanding and digital pathology, as well as recently, drug discovery and vaccine development [199,210]. Accordingly, a systematic review conducted by Carla Pires on the contribution of AI in the development of therapies for COVID-19 concluded that the AI methods accelerate drug repurposing against COVID-19 [211].

To this end, recently, many studies are being conducted to determine the effective drugs against the novel coronavirus, SARS-CoV-2, each of which has proposed some older drugs as effective drugs using different AI models, some of which are currently undergoing clinical trials [210,212–216]. For instance, in an effort, Benevolent AI (London, UK) introduced baricitinib on 4 February 2020, as the potential treatment for COVID-19 by using AI methods [217,218]. Nine months later, baricitinib received emergency use authorization from FDA in hospitalized patients. Since then, a few clinical trials have been conducted to test its effectiveness in improving the clinical status of COVID-19 patients [219–221].

Many companies worldwide have been founded based on AI strategies for drug discovery in recent years. For instance, Evotec (Hamburg, Germany) has started a joint venture with Exscientia (Oxford, UK), an AI-based company, on developing a small molecule, an A2a receptor antagonist, to help T-cell combat solid tumors. This first AI-designed immuno-oncology drug has entered a phase I clinical trial by Evotec. Another candidate drug developed with the help of AI models by Exscientia in partnership with Sumitomo Dainnipon Pharma (Osaka, Japan) is a selective serotonin reuptake inhibitor designed to cure obsessive-compulsive disorder that has entered human clinical trial in Japan. In recent years, many companies, including Benevolent AI, AstraZeneca (Cambridge, UK), Gilead (CA, USA), Insitro (CA, USA), Schrödinger (NY, USA) and so on, have started investing in drug discovery with the help of AI [222].

Although AI and its subdisciplines, such as machine learning and deep learning, have impacted clinical pharmacology in recent years, they also face certain challenges [223]. Some of these challenges include data heterogeneity and low quality, insufficient data shared by companies about candidate drugs or their combinations, and security and interpretability of the models [223,224]. Collectively, despite the problems in the field, AI and its subdisciplines will support the response against COVID-19 in a wide range of areas, including molecular perspectives such as drug discovery and development, clinical perspectives such as diagnosis, clinical outcome prediction and societal perspectives such as epidemiology [225].

## Conclusion

The fact is that the COVID-19 pandemic has disrupted the foundation of healthcare and the economy of our planet. Thus, preventive strategies such as quarantine, dissociation of people in social sites and contact tracing were employed by agencies due to a lack of effective treatment. We reviewed the prophylactic or therapeutic effects of some FDA-approved antiprotozoal and antihelminthic drugs on COVID-19. Despite *in vitro* and *in vivo* success of these drugs, it seems that there still is not any confirmed therapeutic agent for COVID-19.

Even though several millions of doses of vaccines were produced and distributed worldwide, many countries, especially poor ones, still do not have access to it. Vaccines are indeed a good candidate for controlling pandemics like the one the world has been dealing with in the last 2 years; however, the emergence of new variants could affect the effectiveness of vaccines. Furthermore, vaccine preparation is a time-consuming and lengthy process. Hence, we still think there is an urgent need to find strong and effective medications or vaccines that can be prepared easily and inexpensively.

## Future perspective

Considering it is not possible to experimentally test all available drugs in terms of potency against the SARS-CoV-2 in a limited time, leveraging computational methods in the future can speed up this discovery. Machine intelligence methods have revolutionized virtual screening methods for the designing and finding of efficient drugs among massive databases of molecules. AI-based methods, despite some disadvantages, have many advantages, which can lead to their use in a wide range of COVID-19 management aspects. Moreover, it can be applied in all areas of coronavirus research, including virus detection in human samples, detection of the COVID-19 through analyzing medical images, and even the detection of target molecules. For instance, AI technology can be used to scan the viral genome for an effective target locus or target molecule. These new loci and target molecules can then be used not only to find suitable agents among the available drugs but also to design new ones.

**Executive summary**

**Repurposed drugs to harness SARS-CoV-2**

- For controlling the COVID-19 outbreak, there is an urgent need to find new strategies such as antiviral drugs to protect human beings against this disaster.

**Antimalarial drugs**

- Although a large number of antimalarial drugs are under investigation against COVID-19, there is however no certain efficacy reported.

**Ivermectin**

- Ivermectin appears to be effective on COVID-19 and seems to exert efficacy by suppressing the viral protease.

**Artificial intelligence is a state-of-the-art technique to find new drugs**

- Recently, artificial intelligence-based approaches have been increasingly used to increase time and cost-efficiency in finding effective drugs against severe acute respiratory syndrome.
- Molecular docking has emerged as a common tool to discover powerful compounds against SARS-CoV-2.

**Future perspective**

- Even though studies to date have shown the paradoxical effect of anti-parasitic drugs on COVID-19, further investigations with a large-scale population in different geographical areas seem to be necessary.

## References

[1] WHO. COVID-19: One year later – WHO Director-General’s new year message. http://www.who.int/News/Item/30-12-2020-COVID-19-Anniversary-and-Looking-Forward-to-2021

[2] The species Severe acute respiratory syndrome-related coronavirus: classifying 2019-nCoV and naming it SARS-CoV-2. Nat. Microbiol. 5(4), 536–544 (2020). 32123347 10.1038/s41564-020-0695-z PMC7095448

[3] Cheng J, Wang X, Nie T A novel electrochemical sensing platform for detection of dopamine based on gold nanobipyramid/multi-walled carbon nanotube hybrids. Anal. Bioanal. Chem. 412(11), 2433–2441 (2020). 32062832 10.1007/s00216-020-02455-5

[4] Boopathi S, Poma AB, Kolandaivel P. Novel 2019 coronavirus structure, mechanism of action, antiviral drug promises and rule out against its treatment. J. Biomol. Struct. Dyn. 39(9), 3409–3418 (2021). 32306836 10.1080/07391102.2020.1758788 PMC7196923

[5] Hui DSC, Zumla A. Severe acute respiratory syndrome: historical, epidemiologic, and clinical features. Infect. Dis. Clin. North Am. 33(4), 869–889 (2019). 31668196 10.1016/j.idc.2019.07.001 PMC7127569

[6] Guo ZD, Wang ZY, Zhang SF Aerosol and surface distribution of severe acute respiratory syndrome coronavirus 2 in hospital wards, Wuhan, China, 2020. Emerg. Infect. Dis. 26(7), 1583–1591 (2020). 32275497 10.3201/eid2607.200885 PMC7323510

[7] Khateeb J, Li Y, Zhang H. Emerging SARS-CoV-2 variants of concern and potential intervention approaches. Crit. Care 25(1), 244 (2021). 34253247 10.1186/s13054-021-03662-x PMC8274962

[8] Li Z, Nie K, Li K Genome characterization of the first outbreak of COVID-19 Delta Variant B.1.617.2 - Guangzhou City, Guangdong Province, China, May 2021. China CDC Wkly 3(27), 587–589 (2021). 34594942 10.46234/ccdcw2021.151 PMC8392965

[9] Alexandar SRM, Kumar Rs, Jakkan K. . A comprehensive review on COVID-19 Delta variant. Int. J. Pharmacol. Clin. Res. 5, 83–85 (2021).

[10] Planas D, Veyer D, Baidaliuk A Reduced sensitivity of SARS-CoV-2 variant Delta to antibody neutralization. Nature 596(7871), 276–280 (2021). 34237773 10.1038/s41586-021-03777-9

[11] Geng J, Chen L, Yuan Y CD147 antibody specifically and effectively inhibits infection and cytokine storm of SARS-CoV-2 and its variants Delta, Alpha, Beta, and Gamma. Signal. Transduct. Target Ther. 6(1), 347 (2021). 34564690 10.1038/s41392-021-00760-8 PMC8464593

[12] An EUA for sotrovimab for treatment of COVID-19. Med. Lett. Drugs Ther. 63(1627), 97–98 (2021). 34181630

[13] Li M, Lou F, Fan H. SARS-CoV-2 variants of concern Delta: a great challenge to prevention and control of COVID-19. Signal. Transduct. Target Ther. 6(1), 349 (2021). 34580279 10.1038/s41392-021-00767-1 PMC8475295

[14] Desai AN, Majumder MS. What is herd immunity? JAMA 324(20), 2113 (2020). 33074287 10.1001/jama.2020.20895

[15] Papachristodoulou E, Kakoullis L, Parperis K, Panos G. Long-term and herd immunity against SARS-CoV-2: implications from current and past knowledge. Pathog. Dis. 78(3), (2020). 10.1093/femspd/ftaa025 PMC7314002 32510562

[16] Matin S, Talei S, Dalimi A, Dadkhah M, Ghorbani M, Molaei S. COVID-19 and hydatidosis infections: is there any relationship? Iran J. Parasitol. 16(2), 343–345 (2021). 34557252 10.18502/ijpa.v16i2.6287 PMC8418649

[17] Paraskevis D, Kostaki EG, Magiorkinis G, Panayiotakopoulos G, Sourvinos G, Tsiodras S. Full-genome evolutionary analysis of the novel corona virus (2019-nCoV) rejects the hypothesis of emergence as a result of a recent recombination event. Infect. Genet. Evol. 79, 104212 (2020). 32004758 10.1016/j.meegid.2020.104212 PMC7106301

[18] Mousavizadeh L, Ghasemi S. Genotype and phenotype of COVID-19: their roles in pathogenesis. J. Microbiol. Immunol. Infect. 54(2), 159–163 (2021). 32265180 10.1016/j.jmii.2020.03.022 PMC7138183

[19] Chen Y, Liu Q, Guo D. Emerging coronaviruses: genome structure, replication, and pathogenesis. J. Med. Virol. 92(4), 418–423 (2020). 31967327 10.1002/jmv.25681 PMC7167049

[20] Kumar SNR, Maurya VK Saxena SK. Coronavirus disease 2019 (COVID-19), epidemiology, pathogenesis, diagnosis, and therapeutics. In: Morphology, Genome Organization, Replication, and Pathogenesis of Severe Acute Respiratory Syndrome Coronavirus 2 (SARS-CoV-2). Saxena SK (Ed.). Centre for Advanced Research, King George’s Medical University, Lucknow, India, 23–31 (2020).

[21] Wu A, Peng Y, Huang B Genome composition and divergence of the novel coronavirus (2019-nCoV) originating in China. Cell Host Microbe 27(3), 325–328 (2020). 32035028 10.1016/j.chom.2020.02.001 PMC7154514

[22] Perlman S, Netland J. Coronaviruses post-SARS: update on replication and pathogenesis. Nat. Rev. Microbiol. 7(6), 439–450 (2009). 19430490 10.1038/nrmicro2147 PMC2830095

[23] Maier HJ, Bickerton E, Britton P. Preface. Coronaviruses. Methods Mol. Biol. 1282 v (2015). 10.1007/978-1-4939-2438-7 25870870

[24] Wen F, Yu H, Guo J, Li Y, Luo K, Huang S. Identification of the hyper-variable genomic hotspot for the novel coronavirus SARS-CoV-2. J. Infect. 80(6), 671–693 (2020). 10.1016/j.jinf.2020.02.027 PMC7126159 32145215

[25] Lou J, Tian SJ, Niu SM Coronavirus disease 2019: a bibliometric analysis and review. Eur. Rev. Med. Pharmacol. Sci. 24(6), 3411–3421 (2020). 32271460 10.26355/eurrev_202003_20712

[26] Schoeman D, Fielding BC. Coronavirus envelope protein: current knowledge. Virol J. 16(1), 69 (2019). 31133031 10.1186/s12985-019-1182-0 PMC6537279

[27] Salata C, Calistri A, Alvisi G, Celestino M, Parolin C, Palù G. Ebola virus entry: from molecular characterization to drug discovery. Viruses 11(3), 274 (2019). 10.3390/v11030274 PMC6466262 30893774

[28] Delvecchio R, Higa LM, Pezzuto P Chloroquine, an endocytosis blocking agent, inhibits Zika virus infection in different cell models. Viruses 8(12), 322 (2016). 10.3390/v8120322 PMC5192383 27916837

[29] Omotade TO, Roy CR. Manipulation of host cell organelles by intracellular pathogens. Microbiol. Spectr. 7(2), 6484 (2019). 10.1128/microbiolspec.bai-0022-2019 PMC11590418 31025623

[30] Praveen D, Chowdary PR, Aanandhi MV. Janus kinase inhibitor baricitinib is not an ideal option for management of COVID-19. Int. J. Antimicrob. Agents 55(5), 105967 (2020). 32259575 10.1016/j.ijantimicag.2020.105967 PMC7128600

[31] Algaissi A, Hashem AM. Evaluation of MERS-CoV neutralizing antibodies in sera using live virus microneutralization assay. Methods Mol. Biol. 2099, 107–116 (2020). 31883091 10.1007/978-1-0716-0211-9_9 PMC7121888

[32] Goo J, Jeong Y, Park YS Characterization of novel monoclonal antibodies against MERS-coronavirus spike protein. Virus Res. 278, 197863 (2020). 31945421 10.1016/j.virusres.2020.197863 PMC7114870

[33] Pinto D, Park YJ, Beltramello M Cross-neutralization of SARS-CoV-2 by a human monoclonal SARS-CoV antibody. Nature 583(7815), 290–295 (2020). 32422645 10.1038/s41586-020-2349-y

[34] Hijikata A, Shionyu-Mitsuyama C, Nakae S Knowledge-based structural models of SARS-CoV-2 proteins and their complexes with potential drugs. FEBS Lett. 594(12), 1960–1973 (2020). 32379896 10.1002/1873-3468.13806 PMC7267562

[35] Mirza MU, Froeyen M. Structural elucidation of SARS-CoV-2 vital proteins: computational methods reveal potential drug candidates against main protease, Nsp12 polymerase and Nsp13 helicase. J. Pharm. Anal. 10(4), 320–328 (2020). 32346490 10.1016/j.jpha.2020.04.008 PMC7187848

[36] Saha P, Banerjee AK, Tripathi PP, Srivastava AK, Ray U. A virus that has gone viral: amino acid mutation in S protein of Indian isolate of coronavirus COVID-19 might impact receptor binding, and thus, infectivity. Biosci. Rep. 40(5), (2020). 10.1042/BSR20201312 PMC7225408 32378705

[37] Meyer D, Sielaff F, Hammami M, Böttcher-Friebertshäuser E, Garten W, Steinmetzer T. Identification of the first synthetic inhibitors of the type II transmembrane serine protease TMPRSS2 suitable for inhibition of influenza virus activation. Biochem. J. 452(2), 331–343 (2013). 23527573 10.1042/BJ20130101

[38] Wrapp D, Wang N, Corbett KS Cryo-EM structure of the 2019-nCoV spike in the prefusion conformation. Science 367(6483), 1260–1263 (2020). 32075877 10.1126/science.abb2507 PMC7164637

[39] Hoffmann M, Kleine-Weber H, Schroeder S SARS-CoV-2 cell entry depends on ACE2 and TMPRSS2 and is blocked by a clinically proven protease inhibitor. Cell 181(2), 271–280.e278 (2020). 32142651 10.1016/j.cell.2020.02.052 PMC7102627

[40] Rahman N, Basharat Z, Yousuf M, Castaldo G, Rastrelli L, Khan H. Virtual screening of natural products against type II transmembrane serine protease (TMPRSS2), the priming agent of coronavirus 2 (SARS-CoV-2). Molecules 25(10), 2271 (2020). 10.3390/molecules25102271 PMC7287752 32408547

[41] Shirato K, Kawase M, Matsuyama S. Middle East respiratory syndrome coronavirus infection mediated by the transmembrane serine protease TMPRSS2. J. Virol. 87(23), 12552–12561 (2013). 24027332 10.1128/JVI.01890-13 PMC3838146

[42] Sternberg A, Mckee DL, Naujokat C. Novel drugs targeting the SARS-CoV-2/COVID-19 machinery. Curr. Top. Med. Chem. 20(16), 1423–1433 (2020). 32416679 10.2174/1568026620999200517043137

[43] Cao YC, Deng QX, Dai SX. Remdesivir for severe acute respiratory syndrome coronavirus 2 causing COVID-19: an evaluation of the evidence. Travel Med. Infect. Dis. 35, 101647 (2020). 32247927 10.1016/j.tmaid.2020.101647 PMC7151266

[44] Gordon CJ, Tchesnokov EP, Feng JY, Porter DP, Götte M. The antiviral compound remdesivir potently inhibits RNA-dependent RNA polymerase from Middle East respiratory syndrome coronavirus. J. Biol. Chem. 295(15), 4773–4779 (2020). 32094225 10.1074/jbc.AC120.013056 PMC7152756

[45] Mcbride R, Van Zyl M, Fielding BC. The coronavirus nucleocapsid is a multifunctional protein. Viruses 6(8), 2991–3018 (2014). 25105276 10.3390/v6082991 PMC4147684

[46] Sarma P, Shekhar N, Prajapat M In-silico homology assisted identification of inhibitor of RNA binding against 2019-nCoV N-protein (N terminal domain). J. Biomol. Struct. Dyn. 39(8), 2724–2732 (2021). 32266867 10.1080/07391102.2020.1753580 PMC7256351

[47] Bojkova D, Klann K, Koch B Proteomics of SARS-CoV-2-infected host cells reveals therapy targets. Nature 583(7816), 469–472 (2020). 32408336 10.1038/s41586-020-2332-7 PMC7616921

[48] Kirchdoerfer RN, Cottrell CA, Wang N Pre-fusion structure of a human coronavirus spike protein. Nature 531(7592), 118–121 (2016). 26935699 10.1038/nature17200 PMC4860016

[49] Gupta MK, Vemula S, Donde R, Gouda G, Behera L, Vadde R. In-silico approaches to detect inhibitors of the human severe acute respiratory syndrome coronavirus envelope protein ion channel. J. Biomol. Struct. Dyn. 39(7), 2617–2627 (2021). 32238078 10.1080/07391102.2020.1751300 PMC7171389

[50] Lundberg L, Pinkham C, Baer A Nuclear import and export inhibitors alter capsid protein distribution in mammalian cells and reduce Venezuelan Equine Encephalitis Virus replication. Antiviral Res. 100(3), 662–672 (2013). 24161512 10.1016/j.antiviral.2013.10.004

[51] Tu YF, Chien CS, Yarmishyn AA A review of SARS-CoV-2 and the ongoing clinical trials. Int. J. Mol. Sci. 21(7), 2657 (2020). 10.3390/ijms21072657 PMC7177898 32290293

[52] Caly L, Druce JD, Catton MG, Jans DA, Wagstaff KM. The FDA-approved drug ivermectin inhibits the replication of SARS-CoV-2 *in vitro*. Antiviral Res. 178, 104787 (2020). 32251768 10.1016/j.antiviral.2020.104787 PMC7129059

[53] Rezaee H, Pourkarim F, Pourtaghi-Anvarian S, Entezari-Maleki T, Asvadi-Kermani T, Nouri-Vaskeh M. Drug-drug interactions with candidate medications used for COVID-19 treatment: an overview. Pharmacol. Res. Perspect. 9(1), e00705 (2021). 33421347 10.1002/prp2.705 PMC7796804

[54] Talukdar D, Jain V, Balaramnavar V, Srivastava SP, Sivanand P, Gupta MM. Potential drugs for COVID-19 treatment management with their contraindications and drug-drug interaction. Preprints (2021) (Epub ahead of print).

[55] Yang K. What do we know about remdesivir drug interactions? Clin. Transl. Sci. 13(5), 842–844 (2020). 32402130 10.1111/cts.12815 PMC7272915

[56] Kumar D, Trivedi N. Disease-drug and drug-drug interaction in COVID-19: risk and assessment. Biomed. Pharmacother. 139, 111642 (2021). 33940506 10.1016/j.biopha.2021.111642 PMC8078916

[57] Hashem AM, Alghamdi BS, Algaissi AA Therapeutic use of chloroquine and hydroxychloroquine in COVID-19 and other viral infections: a narrative review. Travel Med. Infect. Dis. 35, 101735 (2020). 32387694 10.1016/j.tmaid.2020.101735 PMC7202851

[58] Sachdeva C, Wadhwa A, Kumari A, Hussain F, Jha P, Kaushik NK. *In silico* potential of approved antimalarial drugs for repurposing against COVID-19. Omics 24(10), 568–580 (2020). 32757981 10.1089/omi.2020.0071

[59] Weniger H, World Health Organization. Review of side effects and toxicity of chloroquine / by H. Weniger. (WHO/MAL/79.906) (1979). https://apps.who.int/iris/handle/10665/65773

[60] Goel PGV. Chloroquine. StatPearls Publishing, FL, USA (2019). 31855356

[61] Freedman A, Steinberg VL. Chloroquine in rheumatoid arthritis; a double blindfold trial of treatment for one year. Ann. Rheum. Dis. 19(3), 243–250 (1960). 13701598 10.1136/ard.19.3.243 PMC1007150

[62] Biot C, Daher W, Chavain N Design and synthesis of hydroxyferroquine derivatives with antimalarial and antiviral activities. J. Med. Chem. 49(9), 2845–2849 (2006). 16640347 10.1021/jm0601856

[63] Colson P, Rolain JM, Lagier JC, Brouqui P, Raoult D. Chloroquine and hydroxychloroquine as available weapons to fight COVID-19. Int. J. Antimicrob. Agents 55(4), 105932 (2020). 32145363 10.1016/j.ijantimicag.2020.105932 PMC7135139

[64] Pahan P, Pahan K. Smooth or risky revisit of an old malaria drug for COVID-19? J. Neuroimmune Pharmacol. 15(2), 174–180 (2020). 32415419 10.1007/s11481-020-09923-w PMC7225253

[65] Vincent MJ, Bergeron E, Benjannet S Chloroquine is a potent inhibitor of SARS coronavirus infection and spread. Virol. J. 2, 69 (2005). 16115318 10.1186/1743-422X-2-69 PMC1232869

[66] Wang N, Han S, Liu R Chloroquine and hydroxychloroquine as ACE2 blockers to inhibit viropexis of 2019-nCoV Spike pseudotyped virus. Phytomedicine 79, 153333 (2020). 32920291 10.1016/j.phymed.2020.153333 PMC7467095

[67] Schrezenmeier E, Dörner T. Mechanisms of action of hydroxychloroquine and chloroquine: implications for rheumatology. Nat. Rev. Rheumatol. 16(3), 155–166 (2020). 32034323 10.1038/s41584-020-0372-x

[68] Rainsford KD, Parke AL, Clifford-Rashotte M, Kean WF. Therapy and pharmacological properties of hydroxychloroquine and chloroquine in treatment of systemic lupus erythematosus, rheumatoid arthritis and related diseases. Inflammopharmacology 23(5), 231–269 (2015). 26246395 10.1007/s10787-015-0239-y

[69] Roche PA, Furuta K. The ins and outs of MHC class II-mediated antigen processing and presentation. Nat. Rev. Immunol. 15(4), 203–216 (2015). 25720354 10.1038/nri3818 PMC6314495

[70] Khatooni EBF, Abdi Z. Safety and efficacy of hydroxychloroquine and chloroquine in treatment of COVID-19: a rapid review of evidence. Health Tech. Asmnt. Act 4(1), e5864 (2020).

[71] Pereira BB. Challenges and cares to promote rational use of chloroquine and hydroxychloroquine in the management of coronavirus disease 2019 (COVID-19) pandemic: a timely review. J. Toxicol. Environ. Health B Crit. Rev. 23(4), 177–181 (2020). 32281481 10.1080/10937404.2020.1752340 PMC7157945

[72] Liu J, Cao R, Xu M Hydroxychloroquine, a less toxic derivative of chloroquine, is effective in inhibiting SARS-CoV-2 infection *in vitro*. Cell Discov. 6, 16 (2020). 32194981 10.1038/s41421-020-0156-0 PMC7078228

[73] Naghipour S, Ghodousi M, Rahsepar S, Elyasi S. Repurposing of well-known medications as antivirals: hydroxychloroquine and chloroquine – from HIV-1 infection to COVID-19. Expert Rev. Anti Infect. Ther. 18(11), 1119–1133 (2020). 32631083 10.1080/14787210.2020.1792291

[74] Savarino A, Di Trani L, Donatelli I, Cauda R, Cassone A. New insights into the antiviral effects of chloroquine. Lancet Infect. Dis. 6(2), 67–69 (2006). 16439323 10.1016/S1473-3099(06)70361-9 PMC7129107

[75] Wang M, Cao R, Zhang L Remdesivir and chloroquine effectively inhibit the recently emerged novel coronavirus (2019-nCoV) *in vitro*. Cell Res. 30(3), 269–271 (2020). 32020029 10.1038/s41422-020-0282-0 PMC7054408

[76] Yao X, Ye F, Zhang M *In vitro* antiviral activity and projection of optimized dosing design of hydroxychloroquine for the treatment of severe acute respiratory syndrome coronavirus 2 (SARS-CoV-2). Clin. Infect. Dis. 71(15), 732–739 (2020). 32150618 10.1093/cid/ciaa237 PMC7108130

[77] Huang M, Li M, Xiao F Preliminary evidence from a multicenter prospective observational study of the safety and efficacy of chloroquine for the treatment of COVID-19. Natl Sci. Rev. 7(9), 1428–1436 (2020). 34676087 10.1093/nsr/nwaa113 PMC7313782

[78] Borba MDaVF, Sampaio Vs, Alexandre Ma Chloroquine diphosphate in two different dosages as adjunctive therapy of hospitalized patients with severe respiratory syndrome in the context of coronavirus (SARS-CoV-2) infection: preliminary safety results of a randomized, double-blinded, Phase IIb clinical trial (CloroCOVID-19 study). MedRxiv (2020) (Epub ahead of print).

[79] Lee SH, Son H, Peck KR. Can post-exposure prophylaxis for COVID-19 be considered as an outbreak response strategy in long-term care hospitals? Int. J. Antimicrob. Agents 55(6), 105988 (2020). 32305587 10.1016/j.ijantimicag.2020.105988 PMC7162746

[80] Ullah W, Abdullah HM, Roomi S Safety and efficacy of hydroxychloroquine in COVID-19: a systematic review and meta-analysis. J. Clin. Med. Res. 12(8), 483–491 (2020). 32849936 10.14740/jocmr4233 PMC7430873

[81] Cortegiani A, Ingoglia G, Ippolito M, Giarratano A, Einav S. A systematic review on the efficacy and safety of chloroquine for the treatment of COVID-19. J. Crit. Care 57, 279–283 (2020). 32173110 10.1016/j.jcrc.2020.03.005 PMC7270792

[82] Patil VM, Singhal S, Masand N. A systematic review on use of aminoquinolines for the therapeutic management of COVID-19: efficacy, safety and clinical trials. Life Sci. 254, 117775 (2020). 32418894 10.1016/j.lfs.2020.117775 PMC7211740

[83] Saghir SaM, Algabri NA, Alagawany MM Chloroquine and hydroxychloroquine for the prevention and treatment of COVID-19: a fiction, hope or hype? An updated review. Ther. Clin. Risk Manag. 17, 371–387 (2021). 33953559 10.2147/TCRM.S301817 PMC8092643

[84] Chen J, Liu D, Liu L A pilot study of hydroxychloroquine in treatment of patients with moderate COVID-19. Zhejiang Da Xue Xue Bao Yi Xue Ban 49(2), 215–219 (2020). 32391667 10.3785/j.issn.1008-9292.2020.03.03 PMC8800713

[85] Yu B, Li C, Chen P Low dose of hydroxychloroquine reduces fatality of critically ill patients with COVID-19. Sci. China Life Sci. 63(10), 1515–1521 (2020). 32418114 10.1007/s11427-020-1732-2 PMC7228868

[86] Chowdhury AT, Shahbaz M, Karim R, Islam J, Guo D, He S. A comparative study on ivermectin-doxycycline and hydroxychloroquine-azithromycin therapy on COVID-19 patients. EJMO 5(1), 63–70 (2020).

[87] Gao J, Tian Z, Yang X. Breakthrough: chloroquine phosphate has shown apparent efficacy in treatment of COVID-19 associated pneumonia in clinical studies. Biosci. Trends 14(1), 72–73 (2020). 32074550 10.5582/bst.2020.01047

[88] Spinelli FR, Ceccarelli F, Di Franco M, Conti F. To consider or not antimalarials as a prophylactic intervention in the SARS-CoV-2 (COVID-19) pandemic. Ann. Rheum. Dis. 79(5), 666–667 (2020). 32241791 10.1136/annrheumdis-2020-217367

[89] Chen ZHJ, Zhang Z, Jiang S Efficacy of hydroxychloroquine in patients with COVID-19: results of a randomized clinical trial. Medrxiv (2020) (Epub ahead of print).

[90] Fossa AA, Wisialowski T, Duncan JN, Deng S, Dunne M. Azithromycin/chloroquine combination does not increase cardiac instability despite an increase in monophasic action potential duration in the anesthetized guinea pig. Am. J. Trop. Med. Hyg. 77(5), 929–938 (2007). 17984356

[91] Gautret P, Lagier JC, Parola P Hydroxychloroquine and azithromycin as a treatment of COVID-19: results of an open-label non-randomized clinical trial. Int. J. Antimicrob. Agents 56(1), 105949 (2020). 32205204 10.1016/j.ijantimicag.2020.105949 PMC7102549

[92] Fiolet T, Guihur A, Rebeaud ME, Mulot M, Peiffer-Smadja N, Mahamat-Saleh Y. Effect of hydroxychloroquine with or without azithromycin on the mortality of coronavirus disease 2019 (COVID-19) patients: a systematic review and meta-analysis. Clin. Microbiol. Infect. 27(1), 19–27 (2021). 32860962 10.1016/j.cmi.2020.08.022 PMC7449662

[93] Ghazy RM, Almaghraby A, Shaaban R A systematic review and meta-analysis on chloroquine and hydroxychloroquine as monotherapy or combined with azithromycin in COVID-19 treatment. Sci. Rep. 10(1), 22139 (2020). 33335141 10.1038/s41598-020-77748-x PMC7746770

[94] Geleris J, Sun Y, Platt J Observational study of hydroxychloroquine in hospitalized patients with COVID-19. N. Engl. J. Med. 382(25), 2411–2418 (2020). 32379955 10.1056/NEJMoa2012410 PMC7224609

[95] Rosenberg ES, Dufort EM, Udo T Association of treatment with hydroxychloroquine or azithromycin with in-hospital mortality in patients with COVID-19 in New York state. JAMA 323(24), 2493–2502 (2020). 32392282 10.1001/jama.2020.8630 PMC7215635

[96] Hussein RK, Elkhair HM. Molecular docking identification for the efficacy of some zinc complexes with chloroquine and hydroxychloroquine against main protease of COVID-19. J. Mol. Struct. 1231, 129979 (2021). 33518801 10.1016/j.molstruc.2021.129979 PMC7830318

[97] Chowdhury MS, Rathod J, Gernsheimer J. A rapid systematic review of clinical trials utilizing chloroquine and hydroxychloroquine as a treatment for COVID-19. Acad. Emerg. Med. 27(6), 493–504 (2020). 32359203 10.1111/acem.14005 PMC7267507

[98] Mahévas M, Tran VT, Roumier M No evidence of clinical efficacy of hydroxychloroquine in patients hospitalized for COVID-19 infection with oxygen requirement: results of a study using routinely collected data to emulate a target trial. Medrxiv (2020) (Epub ahead of print).

[99] Mahévas M, Tran VT, Roumier M Clinical efficacy of hydroxychloroquine in patients with COVID-19 pneumonia who require oxygen: observational comparative study using routine care data. BMJ 369, m1844 (2020). 32409486 10.1136/bmj.m1844 PMC7221472

[100] Skipper CP, Pastick KA, Engen NW Hydroxychloroquine in nonhospitalized adults with early COVID-19: a randomized trial. Ann. Intern. Med. 173(8), 623–631 (2020). 32673060 10.7326/M20-4207 PMC7384270

[101] Horby P, Mafham M, Linsell L Effect of hydroxychloroquine in hospitalized patients with COVID-19: preliminary results from a multi-centre, randomized, controlled trial. MedRxiv (2020) (Epub ahead of print).

[102] Patrì A, Fabbrocini G. Hydroxychloroquine and ivermectin: a synergistic combination for COVID-19 chemoprophylaxis and treatment? J. Am. Acad. Dermatol. 82(6), e221 (2020). 32283237 10.1016/j.jaad.2020.04.017 PMC7146719

[103] Mitjà O, Corbacho-Monné M, Ubals M A cluster-randomized trial of hydroxychloroquine for prevention of COVID-19. N. Engl. J. Med. 384(5), 417–427 (2021). 33289973 10.1056/NEJMoa2021801 PMC7722693

[104] Boulware DR, Pullen MF, Bangdiwala AS A randomized trial of hydroxychloroquine as postexposure prophylaxis for COVID-19. N. Engl. J. Med. 383(6), 517–525 (2020). 32492293 10.1056/NEJMoa2016638 PMC7289276

[105] Tang W, Cao Z, Han M Hydroxychloroquine in patients with mainly mild to moderate coronavirus disease 2019: open label, randomised controlled trial. BMJ 369, m1849 (2020). 32409561 10.1136/bmj.m1849 PMC7221473

[106] Kamran SM, Moeed HA, Mirza ZE Clearing the fog: is hydroxychloroquine effective in reducing coronavirus disease-2019 progresression? A randomized controlled trial. Cureus 13(3), e14186 (2021). 33936897 10.7759/cureus.14186 PMC8083993

[107] Ip A, Ahn J, Zhou Y Hydroxychloroquine in the treatment of outpatients with mildly symptomatic COVID-19: a multi-center observational study. BMC Infect. Dis. 21(1), 72 (2021). 33446136 10.1186/s12879-021-05773-w PMC7807228

[108] Oymak Y, Karapinar TH, Devrim İ. Why G6PD deficiency should be screened before COVID-19 treatment with hydroxychloroquine? J. Pediatr. Hematol. Oncol. 43(1), 35–36 (2021). 32496442 10.1097/MPH.0000000000001864

[109] Tleyjeh IM, Kashour Z, Aldosary O Cardiac toxicity of chloroquine or hydroxychloroquine in patients with COVID-19: a systematic review and meta-regression analysis. Mayo Clin. Proc. Innov. Qual. Outcomes 5(1), 137–150 (2021). 33163895 10.1016/j.mayocpiqo.2020.10.005 PMC7605861

[110] Van den Broek MPH, Möhlmann JE, Abeln BGS, Liebregts M, Van Dijk VF, Van De Garde EMW. Chloroquine-induced QTc prolongation in COVID-19 patients. Neth. Heart J. 28(7–8), 406–409 (2020). 32350818 10.1007/s12471-020-01429-7 PMC7189353

[111] Vick DJ. Glucose-6-phosphate dehydrogenase deficiency and COVID-19 infection. Mayo Clin. Proc. 95(8), 1803–1804 (2020). 32680625 10.1016/j.mayocp.2020.05.035 PMC7275177

[112] Chorin E, Wadhwani L, Magnani S QT interval prolongation and torsade de pointes in patients with COVID-19 treated with hydroxychloroquine/azithromycin. Heart Rhythm 17(9), 1425–1433 (2020). 32407884 10.1016/j.hrthm.2020.05.014 PMC7214283

[113] Song Y, Zhang M, Yin L COVID-19 treatment: close to a cure? A rapid review of pharmacotherapies for the novel coronavirus (SARS-CoV-2). Int. J. Antimicrob. Agents 56(2), 106080 (2020). 32634603 10.1016/j.ijantimicag.2020.106080 PMC7334905

[114] WHO. WHO discontinues hydroxychloroquine and lopinavir/ritonavir treatment arms for COVID-19. http://www.who.int/news/item/04-07-2020-who-discontinues-hydroxychloroquine-and-lopinavir-ritonavir-treatment-arms-for-COVID-19

[115] Ho TC, Wang YH, Chen YL Chloroquine and hydroxychloroquine: efficacy in the treatment of the COVID-19. Pathogens 10(2), 217 (2021). 33671315 10.3390/pathogens10020217 PMC7922580

[116] Parperis K. To consider or not antimalarials as a prophylactic intervention in the SARS-CoV-2 (COVID-19) pandemic. Ann. Rheum. Dis. 80(1), e8 (2021). 32321722 10.1136/annrheumdis-2020-217557

[117] Li G, Li Y, Li Z, Zeng M. Artemisinin-based and Other Antimalarials: Detailed Account of Studies by Chinese Scientists Who Discovered and Developed Them. Academic Press London, UK, 1–736 (2017).

[118] Romero MR, Efferth T, Serrano MA Effect of artemisinin/artesunate as inhibitors of hepatitis B virus production in an “*in vitro*” replicative system. Antiviral Res. 68(2), 75–83 (2005). 16122816 10.1016/j.antiviral.2005.07.005

[119] Canivet C, Menasria R, Rhéaume C, Piret J, Boivin G. Valacyclovir combined with artesunate or rapamycin improves the outcome of herpes simplex virus encephalitis in mice compared to antiviral therapy alone. Antiviral Res. 123, 105–113 (2015). 26374952 10.1016/j.antiviral.2015.09.007

[120] Efferth T, Romero MR, Wolf DG, Stamminger T, Marin JJ, Marschall M. The antiviral activities of artemisinin and artesunate. Clin. Infect. Dis. 47(6), 804–811 (2008). 18699744 10.1086/591195

[121] Drouot E, Piret J, Boivin G. Artesunate demonstrates *in vitro* synergism with several antiviral agents against human cytomegalovirus. Antivir. Ther. 21(6), 535–539 (2016). 26844400 10.3851/IMP3028

[122] Dediego ML, Nieto-Torres JL, Regla-Nava JA Inhibition of NF-κB-mediated inflammation in severe acute respiratory syndrome coronavirus-infected mice increases survival. J. Virol. 88(2), 913–924 (2014). 24198408 10.1128/JVI.02576-13 PMC3911641

[123] Lee JY, Bae S, Myoung J. Middle East respiratory syndrome coronavirus-encoded accessory proteins impair MDA5-and TBK1-mediated activation of NF-κB. J. Microbiol. Biotechnol. 29(8), 1316–1323 (2019). 31434175 10.4014/jmb.1908.08004

[124] Brian DA, Baric RS. Coronavirus genome structure and replication. Curr. Top. Microbiol. Immunol. 287, 1–30 (2005). 15609507 10.1007/3-540-26765-4_1 PMC7120446

[125] Catanzaro M, Fagiani F, Racchi M, Corsini E, Govoni S, Lanni C. Immune response in COVID-19: addressing a pharmacological challenge by targeting pathways triggered by SARS-CoV-2. Signal. Transduct. Target Ther. 5(1), 84 (2020). 32467561 10.1038/s41392-020-0191-1 PMC7255975

[126] Xu H, He Y, Yang X Anti-malarial agent artesunate inhibits TNF-alpha-induced production of proinflammatory cytokines via inhibition of NF-kappaB and PI3 kinase/Akt signal pathway in human rheumatoid arthritis fibroblast-like synoviocytes. Rheumatology (Oxford) 46(6), 920–926 (2007). 17314215 10.1093/rheumatology/kem014

[127] Moore JB, June CH. Cytokine release syndrome in severe COVID-19. Science 368(6490), 473–474 (2020). 32303591 10.1126/science.abb8925

[128] Cao R, Hu H, Li Y Anti-SARS-CoV-2 potential of artemisinins *in vitro*. ACS Infect. Dis. 6(9), 2524–2531 (2020). 32786284 10.1021/acsinfecdis.0c00522

[129] Nie C, Trimpert J, Moon S *In vitro* efficacy of Artemisia extracts against SARS-CoV-2. Virol. J. 18(1), 182 (2021). 34496903 10.1186/s12985-021-01651-8 PMC8424155

[130] Russo M, Moccia S, Spagnuolo C, Tedesco I, Russo GL. Roles of flavonoids against coronavirus infection. Chem. Biol. Interact. 328, 109211 (2020). 32735799 10.1016/j.cbi.2020.109211 PMC7385538

[131] Zhou Y, Gilmore K, Ramirez S *In vitro* efficacy of artemisinin-based treatments against SARS-CoV-2. Sci. Rep. 11(1), 14571 (2021). 34272426 10.1038/s41598-021-93361-y PMC8285423

[132] Nair MS, Huang Y, Fidock DA *Artemisia annua L.* extracts inhibit the *in vitro* replication of SARS-CoV-2 and two of its variants. J. Ethnopharmacol. 274, 114016 (2021). 33716085 10.1016/j.jep.2021.114016 PMC7952131

[133] Hu Y, Liu M, Qin H Artemether, artesunate, arteannuin B, echinatin, licochalcone B and andrographolide effectively inhibit SARS-CoV-2 and related viruses *in vitro*. Front. Cell. Infect. Microbiol. 11, 680127 (2021). 34527599 10.3389/fcimb.2021.680127 PMC8435859

[134] Krishna S, Augustin Y, Wang J Repurposing antimalarials to tackle the COVID-19 pandemic. Trends Parasitol. 37(1), 8–11 (2021). 33153922 10.1016/j.pt.2020.10.003 PMC7572038

[135] Uckun FM, Saund S, Windlass H, Trieu V. Repurposing anti-malaria phytomedicine artemisinin as a COVID-19 drug. Front. Pharmacol. 12, 649532 (2021). 33815126 10.3389/fphar.2021.649532 PMC8017220

[136] Fry MPM. Site of action of the antimalarial hydroxynaphthoquinone, 2-[trans-4-(4’-chlorophenyl) cyclohexyl]-3-hydroxy-1, 4-naphthoquinone (566C80). Biochem. Pharmacol. 43(7), 1545–1553 (1992). 1314606 10.1016/0006-2952(92)90213-3

[137] Atovaquone and azithromycin combination for confirmed COVID-19 infection. NCT04339426. https://clinicaltrials.gov/ct2/show/NCT04339426

[138] Singh S, Florez H. Coronavirus disease 2019 drug discovery through molecular docking. F1000Res 9, 502 (2020). 32704354 10.12688/f1000research.24218.1 PMC7361499

[139] Farag A, Wang P, Boys In Identification of atovaquone, ouabain and mebendazole as FDA approved drugs targeting SARS-CoV-2 (version 4). ChemRxiv (2021) (Epub ahead of print).

[140] Marak BN, Dowarah J, Khiangte L, Singh VP. Step toward repurposing drug discovery for COVID-19 therapeutics through *in silico* approach. Drug Dev. Res. 82(3), 374–392 (2021). 33170521 10.1002/ddr.21757

[141] Carter-Timofte ME, Arulanandam R, Kurmasheva N Antiviral potential of the antimicrobial drug atovaquone against SARS-CoV-2 and emerging variants of concern. ACS Infect. Dis. 7(11), 3034–3051 (2021). 34658235 10.1021/acsinfecdis.1c00278

[142] González Canga A, Sahagún Prieto AM, Diez Liébana MJ, Fernández Martínez N, Sierra Vega M, García Vieitez JJ. The pharmacokinetics and interactions of ivermectin in humans – a mini-review. AAPS J 10(1), 42–46 (2008). 18446504 10.1208/s12248-007-9000-9 PMC2751445

[143] Laing R, Gillan V, Devaney E. Ivermectin - old drug, new tricks? Trends Parasitol. 33(6), 463–472 (2017). 28285851 10.1016/j.pt.2017.02.004 PMC5446326

[144] Azam F, Taban IM, Eid EEM An in-silico analysis of ivermectin interaction with potential SARS-CoV-2 targets and host nuclear importin α. J. Biomol. Struct. Dyn., 1–14 (2020) (Epub ahead of print). 10.1080/07391102.2020.1841028 PMC7643422 33131430

[145] Azeem S, Ashraf M, Rasheed MA, Anjum AA, Hameed R. Evaluation of cytotoxicity and antiviral activity of ivermectin against Newcastle disease virus. Pak. J. Pharm. Sci. 28(2), 597–602 (2015). 25730813

[146] Chaccour C, Hammann F, Ramón-García S, Rabinovich NR. Ivermectin and COVID-19: keeping rigor in times of urgency. Am. J. Trop. Med. Hyg. 102(6), 1156–1157 (2020). 32314704 10.4269/ajtmh.20-0271 PMC7253113

[147] Götz V, Magar L, Dornfeld D Influenza A viruses escape from MxA restriction at the expense of efficient nuclear vRNP import. Sci. Rep. 6, 23138 (2016). 26988202 10.1038/srep23138 PMC4796820

[148] Lv C, Liu W, Wang B Ivermectin inhibits DNA polymerase UL42 of pseudorabies virus entrance into the nucleus and proliferation of the virus *in vitro* and vivo. Antiviral Res. 159, 55–62 (2018). 30266338 10.1016/j.antiviral.2018.09.010

[149] Rizzo E. Ivermectin, antiviral properties and COVID-19: a possible new mechanism of action. Naunyn Schmiedebergs Arch. Pharmacol. 393(7), 1153–1156 (2020). 32462282 10.1007/s00210-020-01902-5 PMC7251046

[150] Gupta D, Sahoo AK, Singh A. Ivermectin: potential candidate for the treatment of Covid 19. Braz. J. Infect. Dis. 24(4), 369–371 (2020). 32615072 10.1016/j.bjid.2020.06.002 PMC7321032

[151] Hashim HA, Maulood MF, Rasheed AM, Fatak DF, Kabah KK, Abdulamir AS. Controlled randomized clinical trial on using Ivermectin with doxycycline for treating COVID-19 patients in Baghdad, Iraq. MedRxiv (2020) (Epub ahead of print).

[152] Rajter JC, Sherman MS, Fatteh N, Vogel F, Sacks J, Rajter JJ. Use of ivermectin is associated with lower mortality in hospitalized patients with coronavirus disease 2019: the ivermectin in COVID nineteen study. Chest 159(1), 85–92 (2021). 33065103 10.1016/j.chest.2020.10.009 PMC7550891

[153] Rajter JC, Sherman MS, Fatteh N, Vogel F, Sacks J, Rajter JJ. ICON (ivermectin in covid nineteen) study: use of ivermectin is associated with lower mortality in hospitalized patients with COVID-19. Chest 159(1), 85–92 (2021). 33065103 10.1016/j.chest.2020.10.009 PMC7550891

[154] Ahmed S, Karim MM, Ross AG A five-day course of ivermectin for the treatment of COVID-19 may reduce the duration of illness. Int. J. Infect. Dis. 103, 214–216 (2021). 33278625 10.1016/j.ijid.2020.11.191 PMC7709596

[155] López-Medina E, López P, Hurtado IC Effect of ivermectin on time to resolution of symptoms among adults with mild COVID-19: a randomized clinical trial. JAMA 325(14), 1426–1435 (2021). 33662102 10.1001/jama.2021.3071 PMC7934083

[156] Bryant A, Lawrie TA, Dowswell T Ivermectin for prevention and treatment of COVID-19 infection: a systematic review, meta-analysis, and trial sequential analysis to inform clinical guidelines. Am. J. Ther. 28(4), e434–e460 (2021). 34145166 10.1097/MJT.0000000000001402 PMC8248252

[157] Kaur H, Shekhar N, Sharma S, Sarma P, Prakash A, Medhi B. Ivermectin as a potential drug for treatment of COVID-19: an in-sync review with clinical and computational attributes. Pharmacol. Rep. 73(3), 736–749 (2021). 33389725 10.1007/s43440-020-00195-y PMC7778723

[158] Ivermectin for COVID-19: real-time meta analysis of 65 studies. https://ivmmeta.com

[159] Altay O, Mohammadi E, Lam S Current Status of COVID-19 therapies and drug repositioning applications. iScience 23(7), 101303 (2020). 32622261 10.1016/j.isci.2020.101303 PMC7305759

[160] Haffizulla J, Hartman A, Hoppers M Effect of nitazoxanide in adults and adolescents with acute uncomplicated influenza: a double-blind, randomised, placebo-controlled, phase IIb/III trial. Lancet Infect. Dis. 14(7), 609–618 (2014). 24852376 10.1016/S1473-3099(14)70717-0 PMC7164783

[161] Rossignol JF. Nitazoxanide: a first-in-class broad-spectrum antiviral agent. Antiviral Res. 110, 94–103 (2014). 25108173 10.1016/j.antiviral.2014.07.014 PMC7113776

[162] Tilmanis D, Van Baalen C, Oh DY, Rossignol JF, Hurt AC. The susceptibility of circulating human influenza viruses to tizoxanide, the active metabolite of nitazoxanide. Antiviral Res. 147, 142–148 (2017). 28986103 10.1016/j.antiviral.2017.10.002

[163] Rossignol JF. Nitazoxanide, a new drug candidate for the treatment of Middle East respiratory syndrome coronavirus. J. Infect. Public Health 9(3), 227–230 (2016). 27095301 10.1016/j.jiph.2016.04.001 PMC7102735

[164] Calderón JM, Zerón HM, Padmanabhan S. Treatment with hydroxychloroquine vs hydroxychloroquine + nitazoxanide in COVID-19 patients with risk factors for poor prognosis: a structured summary of a study protocol for a randomised controlled trial. Trials 21(1), 504 (2020). 32513231 10.1186/s13063-020-04448-2 PMC7276957

[165] Padmanabhan S, Padmanabhan K. “The devil is in the dosing” – targeting the interferon pathway by repositioning nitazoxanide against COVID-19. (2020) (Epub ahead of print).

[166] Hong SK, Kim HJ, Song CS, Choi IS, Lee JB, Park SY. Nitazoxanide suppresses IL-6 production in LPS-stimulated mouse macrophages and TG-injected mice. Int. Immunopharmacol. 13(1), 23–27 (2012). 22430099 10.1016/j.intimp.2012.03.002

[167] Mehta P, Mcauley DF, Brown M, Sanchez E, Tattersall RS, Manson JJ. COVID-19: consider cytokine storm syndromes and immunosuppression. Lancet 395(10229), 1033–1034 (2020). 32192578 10.1016/S0140-6736(20)30628-0 PMC7270045

[168] Miner K, Labitzke K, Liu B Drug repurposing: the anthelmintics niclosamide and nitazoxanide are potent TMEM16A antagonists that fully bronchodilate airways. Front. Pharmacol. 10, 51 (2019). 30837866 10.3389/fphar.2019.00051 PMC6382696

[169] Rocco PRM, Silva PL, Cruz FF Early use of nitazoxanide in mild COVID-19 disease: randomised, placebo-controlled trial. Eur. Respir. J. 58(1), 2003725 (2021). 33361100 10.1183/13993003.03725-2020 PMC7758778

[170] Elalfy H, Besheer T, El-Mesery A Effect of a combination of nitazoxanide, ribavirin, and ivermectin plus zinc supplement (MANS.NRIZ study) on the clearance of mild COVID-19. J. Med. Virol. 93(5), 3176–3183 (2021). 33590901 10.1002/jmv.26880 PMC8014583

[171] Kelleni MT. NSAIDs and Kelleni’s protocol as potential early COVID-19 treatment game changer: could it be the final countdown? .Inflammopharmacology , 1–6 (2021) (Epub ahead of print). 34822026 10.1007/s10787-021-00896-7 PMC8613510

[172] Pepperrell T, Pilkington V, Owen A, Wang J, Hill AM. Review of safety and minimum pricing of nitazoxanide for potential treatment of COVID-19. J. Virus Erad. 6(2), 52–60 (2020). 32405422 10.1016/S2055-6640(20)30017-0 PMC7332204

[173] Rajoli RKR, Pertinez H, Arshad U Dose prediction for repurposing nitazoxanide in SARS-CoV-2 treatment or chemoprophylaxis. Br. J. Clin. Pharmacol. 87(4), 2078–2088 (2021). 33085781 10.1111/bcp.14619 PMC8056737

[174] Blum VF, Cimerman S, Hunter JR Nitazoxanide superiority to placebo to treat moderate COVID-19 – a pilot prove of concept randomized double-blind clinical trial. EClinicalMedicine 37, 100981 (2021). 34222847 10.1016/j.eclinm.2021.100981 PMC8235996

[175] Cadegiani FA, Goren A, Wambier CG, Mccoy J. Early COVID-19 therapy with azithromycin plus nitazoxanide, ivermectin or hydroxychloroquine in outpatient settings significantly improved COVID-19 outcomes compared to known outcomes in untreated patients. New Microbes New Infect. 43, 100915 (2021). 34249367 10.1016/j.nmni.2021.100915 PMC8262389

[176] Olagunju A, Fowotade A, Olagunoye A Efficacy and safety of nitazoxanide plus atazanavir/ritonavir for the treatment of moderate to severe COVID-19 (NACOVID): a structured summary of a study protocol for a randomised controlled trial. Trials 22(1), 3 (2021). 33397457 10.1186/s13063-020-04987-8 PMC7780204

[177] Sayed AM, Khalaf AM, Abdelrahim MEA, Elgendy MO. Repurposing of some anti-infective drugs for COVID-19 treatment: a surveillance study supported by an *in silico* investigation. Int. J. Clin. Pract. 75(4), e13877 (2021). 33300221 10.1111/ijcp.13877 PMC7883047

[178] MendietaZerón H, Meneses Calderón J, Paniagua Coria L Nitazoxanide as an early treatment to reduce the intensity of COVID-19 outbreaks among health personnel. World Acad. Sci. J. 3(3), 23 (2021).

[179] Efficacy of Nitazoxanide in reducing the viral load in COVID-19 patients. Randomized, placebo-controlled, single-blinded, parallel group, pilot study. MedRxiv (2021) (Epub ahead of print).

[180] Chen W, Mook RA Jr, Premont RT, Wang J. Niclosamide: beyond an antihelminthic drug. Cell. Signal. 41, 89–96 (2018). 28389414 10.1016/j.cellsig.2017.04.001 PMC5628105

[181] Xu M, Lee EM, Wen Z Identification of small-molecule inhibitors of Zika virus infection and induced neural cell death via a drug repurposing screen. Nat. Med. 22(10), 1101–1107 (2016). 27571349 10.1038/nm.4184 PMC5386783

[182] Wu CJ, Jan JT, Chen CM Inhibition of severe acute respiratory syndrome coronavirus replication by niclosamide. Antimicrob. Agents Chemother. 48(7), 2693–2696 (2004). 15215127 10.1128/AAC.48.7.2693-2696.2004 PMC434198

[183] Xu J, Shi PY, Li H, Zhou J. Broad spectrum antiviral agent niclosamide and its therapeutic potential. ACS Infect. Dis. 6(5), 909–915 (2020). 32125140 10.1021/acsinfecdis.0c00052 PMC7098069

[184] Ko M, Chang SY, Byun SY Screening of FDA-approved drugs using a MERS-CoV clinical isolate from South Korea identifies potential therapeutic options for COVID-19. Viruses 13(4), 651(2021). 33918958 10.3390/v13040651 PMC8069929

[185] Romani D, Noureddine O, Issaoui N, Brandán SA. Properties and reactivities of niclosamide in different media, a potential antiviral to treatment of COVID-19 by using DFT calculations and molecular docking. Biointerface Res. Appl. Chem 10(6), 7295–7328 (2020).

[186] Gao K, Nguyen DD, Chen J, Wang R, Wei GW. Repositioning of 8565 existing drugs for COVID-19. J. Phys. Chem. Lett. 11(13), 5373–5382 (2020). 32543196 10.1021/acs.jpclett.0c01579 PMC7313673

[187] Pindiprolu S, Pindiprolu SH. Plausible mechanisms of niclosamide as an antiviral agent against COVID-19. Med. Hypotheses 140, 109765 (2020). 32361588 10.1016/j.mehy.2020.109765 PMC7195103

[188] Gassen NC, Papies J, Bajaj T Analysis of SARS-CoV-2-controlled autophagy reveals spermidine, MK-2206, and niclosamide as putative antiviral therapeutics. BioRxiv (2020) (Epub ahead of print).

[189] Das S, Sarmah S, Lyndem S, Singha Roy A. An investigation into the identification of potential inhibitors of SARS-CoV-2 main protease using molecular docking study. J. Biomol. Struct. Dyn. 39(9), 3347–3357 (2021). 32362245 10.1080/07391102.2020.1763201 PMC7232884

[190] Backer V, Sjöbring U, Sonne J A randomized, double-blind, placebo-controlled phase I trial of inhaled and intranasal niclosamide: a broad spectrum antiviral candidate for treatment of COVID-19. Lancet Reg. Health Eur. 4, 100084 (2021). 33842908 10.1016/j.lanepe.2021.100084 PMC8021896

[191] Pascoalino BS, Courtemanche G, Cordeiro MT, Gil LH, Freitas-Junior L. Zika antiviral chemotherapy: identification of drugs and promising starting points for drug discovery from an FDA-approved library. F1000Res 5, 2523 (2016). 27909576 10.12688/f1000research.9648.1 PMC5112578

[192] Tonelli M, Simone M, Tasso B Antiviral activity of benzimidazole derivatives. II. Antiviral activity of 2-phenylbenzimidazole derivatives. Bioorg. Med. Chem. 18(8), 2937–2953 (2010). 20359898 10.1016/j.bmc.2010.02.037

[193] Farag A, Wang P, Boys IN Identification of atovaquone, ouabain and mebendazole as FDA approved drugs targeting SARS-CoV-2 (Version 4). Chemrxiv (2020) (Epub ahead of print).

[194] Wang Z, Guo K, Gao P Identification of repurposable drugs and adverse drug reactions for various courses of COVID-19 based on single-cell RNA sequencing data. ArXiv, (2020) (Epub ahead of print).

[195] Law JN, Akers K, Tasnina N Interpretable network propagation with application to expanding the repertoire of human proteins that interact with SARS-CoV-2. GigaSci. 10(12), (2021) (Epub ahead of print). 10.1093/gigascience/giab082 PMC8716363 34966926

[196] Hajjo R, Tropsha A. A systems biology workflow for drug and vaccine repurposing: identifying small-molecule BCG mimics to reduce or prevent COVID-19 mortality. Pharm. Res. 37(11), 212 (2020). 33025261 10.1007/s11095-020-02930-9 PMC7537965

[197] Repurposed antiviral drugs for COVID-19 – interim WHO solidarity trial results. N. Engl. J. Med. 384(6), 497–511 (2020). 33264556 10.1056/NEJMoa2023184 PMC7727327

[198] Chan HCS, Shan H, Dahoun T, Vogel H, Yuan S. Advancing drug discovery via artificial intelligence. Trends Pharmacol. Sci. 40(8), 592–604 (2019). 31320117 10.1016/j.tips.2019.06.004

[199] Alsafi MA, Hughes DL, Said MA. First COVID-19 molecular docking with a chalcone-based compound: synthesis, single-crystal structure and Hirshfeld surface analysis study. Acta Crystallogr. C Struct. Chem. 76(Pt 12), 1043–1050 (2020). 33273140 10.1107/S2053229620014217

[200] Barros RO, Junior F, Pereira WS, Oliveira NMN, Ramos RM. Interaction of drug candidates with various SARS-CoV-2 receptors: an *in silico* study to combat COVID-19. J. Proteome Res. 19(11), 4567–4575 (2020). 32786890 10.1021/acs.jproteome.0c00327

[201] Hall DC Jr, Ji HF. A search for medications to treat COVID-19 via *in silico* molecular docking models of the SARS-CoV-2 spike glycoprotein and 3CL protease. Travel Med. Infect. Dis. 35, 101646 (2020). 32294562 10.1016/j.tmaid.2020.101646 PMC7152904

[202] Keshavarzi Arshadi A, Webb J, Salem M Artificial intelligence for COVID-19 drug discovery and vaccine development. Front. Artif. Intell. 3, 65 (2020). 33733182 10.3389/frai.2020.00065 PMC7861281

[203] Peele KA, Potla Durthi C, Srihansa T Molecular docking and dynamic simulations for antiviral compounds against SARS-CoV-2: a computational study. Inform. Med. Unlocked 19, 100345 (2020). 32395606 10.1016/j.imu.2020.100345 PMC7211761

[204] Podlogar BL, Muegge I, Brice LJ. Computational methods to estimate drug development parameters. Curr. Opin. Drug Discov. Devel. 4(1), 102–109 (2001). 11727315

[205] Lestari K, Sitorus T, Instiaty Megantara S, Levita J. Molecular docking of quinine, chloroquine and hydroxychloroquine to angiotensin converting enzyme 2 (ACE2) receptor for discovering new potential COVID-19 antidote. J. Adv. Pharm. Res. 10(2), 1–4 (2020).

[206] Shoichet BK. Virtual screening of chemical libraries. Nature 432(7019), 862–865 (2004). 15602552 10.1038/nature03197 PMC1360234

[207] Melville JL, Burke EK, Hirst JD. Machine learning in virtual screening. Comb. Chem. High Throughput Screen 12(4), 332–343 (2009). 19442063 10.2174/138620709788167980

[208] Carpenter KA, Huang X. Machine learning-based virtual screening and its applications to Alzheimer’s drug discovery: a review. Curr. Pharm. Des. 24(28), 3347–3358 (2018). 29879881 10.2174/1381612824666180607124038 PMC6327115

[209] Zhong F, Xing J, Li X Artificial intelligence in drug design. Sci. China Life Sci. 61(10), 1191–1204 (2018). 30054833 10.1007/s11427-018-9342-2

[210] Ahuja AS, Reddy VP, Marques O. Artificial intelligence and COVID-19: a multidisciplinary approach. Integr. Med. Res. 9(3), 100434 (2020). 32632356 10.1016/j.imr.2020.100434 PMC7255319

[211] Pires C. A systematic review on the contribution of artificial intelligence in the development of medicines for COVID-2019. J. Pers. Med. 11(9), (2021). 10.3390/jpm11090926 PMC8465965 34575703

[212] Ke YY, Peng TT, Yeh TK Artificial intelligence approach fighting COVID-19 with repurposing drugs. Biomed. J. 43(4), 355–362 (2020). 32426387 10.1016/j.bj.2020.05.001 PMC7227517

[213] Kowalewski J, Ray A. Predicting novel drugs for SARS-CoV-2 using machine learning from a >10 million chemical space. Heliyon 6(8), e04639 (2020). 32802980 10.1016/j.heliyon.2020.e04639 PMC7409807

[214] Avchaciov K, Burmistrova O, Fedichev P. AI for the repurposing of approved or investigational drugs against COVID-19. ScienceOPEN (2020) (Epub ahead of print).

[215] Gysi Dm DVÍ, Zitnik M Proceedings of the national academy of sciences network medicine framework for identifying drug-repurposing opportunities for COVID-19. Proc. Natl Acad. Sci. 118(19), (2021). 10.1073/pnas.2025581118 PMC8126852 33906951

[216] Moskal M, Beker W, Roszak R Suggestions for second-pass anti-COVID-19 drugs based on the artificial intelligence measures of molecular similarity, shape and pharmacophore distribution. ChemRxiv (2020) (Epub ahead of print).

[217] Richardson P, Griffin I, Tucker C Baricitinib as potential treatment for 2019-nCoV acute respiratory disease. Lancet 395(10223), e30–e31 (2020). 32032529 10.1016/S0140-6736(20)30304-4 PMC7137985

[218] Stebbing J, Phelan A, Griffin I COVID-19: combining antiviral and anti-inflammatory treatments. Lancet Infect. Dis. 20(4), 400–402 (2020). 32113509 10.1016/S1473-3099(20)30132-8 PMC7158903

[219] Kalil AC, Patterson TF, Mehta AK Baricitinib plus remdesivir for hospitalized adults with COVID-19. N. Engl. J. Med. 384(9), 795–807 (2021). 33306283 10.1056/NEJMoa2031994 PMC7745180

[220] Marconi VC, Ramanan AV, De Bono S Efficacy and safety of baricitinib for the treatment of hospitalised adults with COVID-19 (COV-BARRIER): a randomised, double-blind, parallel-group, placebo-controlled phase III trial. Lancet Respir. Med. 9(12), 1407–1418 (2021). 34480861 10.1016/S2213-2600(21)00331-3 PMC8409066

[221] Stebbing J, Sánchez Nievas G, Falcone M JAK inhibition reduces SARS-CoV-2 liver infectivity and modulates inflammatory responses to reduce morbidity and mortality. Sci. Adv. 7(1), eabe4724 (2021). 33187978 10.1126/sciadv.abe4724 PMC7775747

[222] Savage N. Tapping into the drug discovery potential of AI. http://www.nature.com/articles/d43747-021-00045-7

[223] Zhavoronkov A, Vanhaelen Q, Oprea TI. Will artificial intelligence for drug discovery impact clinical pharmacology? Clin. Pharmacol. Ther. 107(4), 780–785 (2020). 31957003 10.1002/cpt.1795 PMC7158211

[224] Bhhatarai B, Walters WP, Hop C, Lanza G, Ekins S. Opportunities and challenges using artificial intelligence in ADME/Tox. Nat. Mater. 18(5), 418–422 (2019). 31000801 10.1038/s41563-019-0332-5 PMC6594826

[225] Bullock J, Luccioni A, Pham KH, Lam CSN, Luengo-Oroz M. Mapping the landscape of artificial intelligence applications against COVID-19. J. Artif. Intell. Res. 69, 807–845 (2020).

